# Mesenchymal-to-epithelial transitions require tissue-specific interactions with distinct laminins

**DOI:** 10.1083/jcb.202010154

**Published:** 2021-05-28

**Authors:** Ioanna Pitsidianaki, Jason Morgan, Jamie Adams, Kyra Campbell

**Affiliations:** Department of Biomedical Science and Bateson Centre, The University of Sheffield, Sheffield, UK

## Abstract

Mesenchymal-to-epithelial transition (MET) converts cells from migratory mesenchymal to polarized epithelial states. Despite its importance for both normal and pathological processes, very little is known about the regulation of MET in vivo*.* Here we exploit midgut morphogenesis in *Drosophila melanogaster* to investigate the mechanisms underlying MET. We show that down-regulation of the EMT transcription factor Serpent is required for MET, but not sufficient, as interactions with the surrounding mesoderm are also essential. We find that midgut MET relies on the secretion of specific laminins via the CopII secretory pathway from both mesoderm and midgut cells. We show that secretion of the laminin trimer containing the Wingblister α-subunit from the mesoderm is an upstream cue for midgut MET, leading to basal polarization of αPS1 integrin in midgut cells. Polarized αPS1 is required for the formation of a monolayered columnar epithelium and for the apical polarization of αPS3, Baz, and E-Cad. Secretion of a distinct LamininA-containing trimer from midgut cells is required to reinforce the localization of αPS1 basally, and αPS3 apically, for robust repolarization. Our data suggest that targeting these MET pathways, in conjunction with therapies preventing EMT, may present a two-pronged strategy toward blocking metastasis in cancer.

## Teaser

Pitsidianaki et al. show that the mesenchymal-to-epithelial transition in the *Drosophila melanogaster* midgut relies both on downregulation of the EMT-transcription factor Serpent and on the secretion of a specific laminin trimer from the neighboring mesoderm.

## Introduction

The ability of epithelial cells to reversibly transition toward mesenchymal states is crucial for the formation of many tissues and organs during development, and is also a key driver of cancer metastasis (Nieto et al., 2016; Plygawko et al., 2020). This plasticity involves cells undergoing epithelial-to-mesenchymal transitions (EMTs), which facilitates cell migration and invasion, and the far less well-characterized mesenchymal-to-epithelial transition (MET), where cells convert toward a more stationary, epithelial phenotype. MET is employed during epithelial development and is characterized by the progressive establishment of apicobasal cell polarity (Pei et al., 2019). MET also appears to play a central role in metastatic colonization, where mesenchymal tumor cells revert to a more epithelial state via MET in order to proliferate and form secondary growths in distant organs (Yang et al., 2020).

While many upstream regulators of EMT, including EMT transcription factors (TFs), have been identified, very little is known about the regulation of MET, and in particular how it relates to EMT. Available information is largely based on in vitro analysis or animal models in which MET is triggered by forced down-regulation of an EMT-TF to study cancer metastasis (Ocaña et al., 2012; Tran et al., 2014; Tsai et al., 2012). Such studies have led to the hypothesis that EMT-TF down-regulation is sufficient for MET to occur (Dongre and Weinberg, 2019). This has important implications, as it suggests that therapeutic strategies aimed at blocking EMT to prevent delamination from primary tumors may in fact favor MET in cancer cells. This favoring of MET may inadvertently promote secondary metastasis formation by cells that have already disseminated into the bloodstream. It is therefore critical to understand the regulation of MET in vivo*,* and how the underlying mechanisms relate to those driving EMT.

Here we leverage the *Drosophila melanogaster* embryonic midgut, a powerful model for studying epithelial-mesenchymal plasticity, and show that while the down-regulation of an EMT-TF is required for MET, it is not sufficient. In addition to cell-intrinsic changes, highly specific interactions with the surrounding environment are required. We show that upon down-regulation of the EMT-TF Serpent, midgut cells repolarize in response to the tissue-specific secretion of the laminin trimer containing the vertebrate α1,2 laminin homologue, *wing blister* (*wb*)*.* This demonstrates, in an in vivo context, that the down-regulation of an EMT-TF and MET are genetically separable events. Furthermore, the requirement for extrinsic signals may explain why cells only undergo MET in certain regions of the developing embryo, or why cancer cells undergoing MET invade specific tissues. Finally, combining therapies that prevent EMT with those that target specific ECM components linked to metastatic MET presents a promising new strategy for treating metastatic cancer, averting the potentially detrimental effects of blocking EMT alone.

## Results

### Midgut MET requires the down-regulation of the EMT-TF Serpent

**Figure S1.**
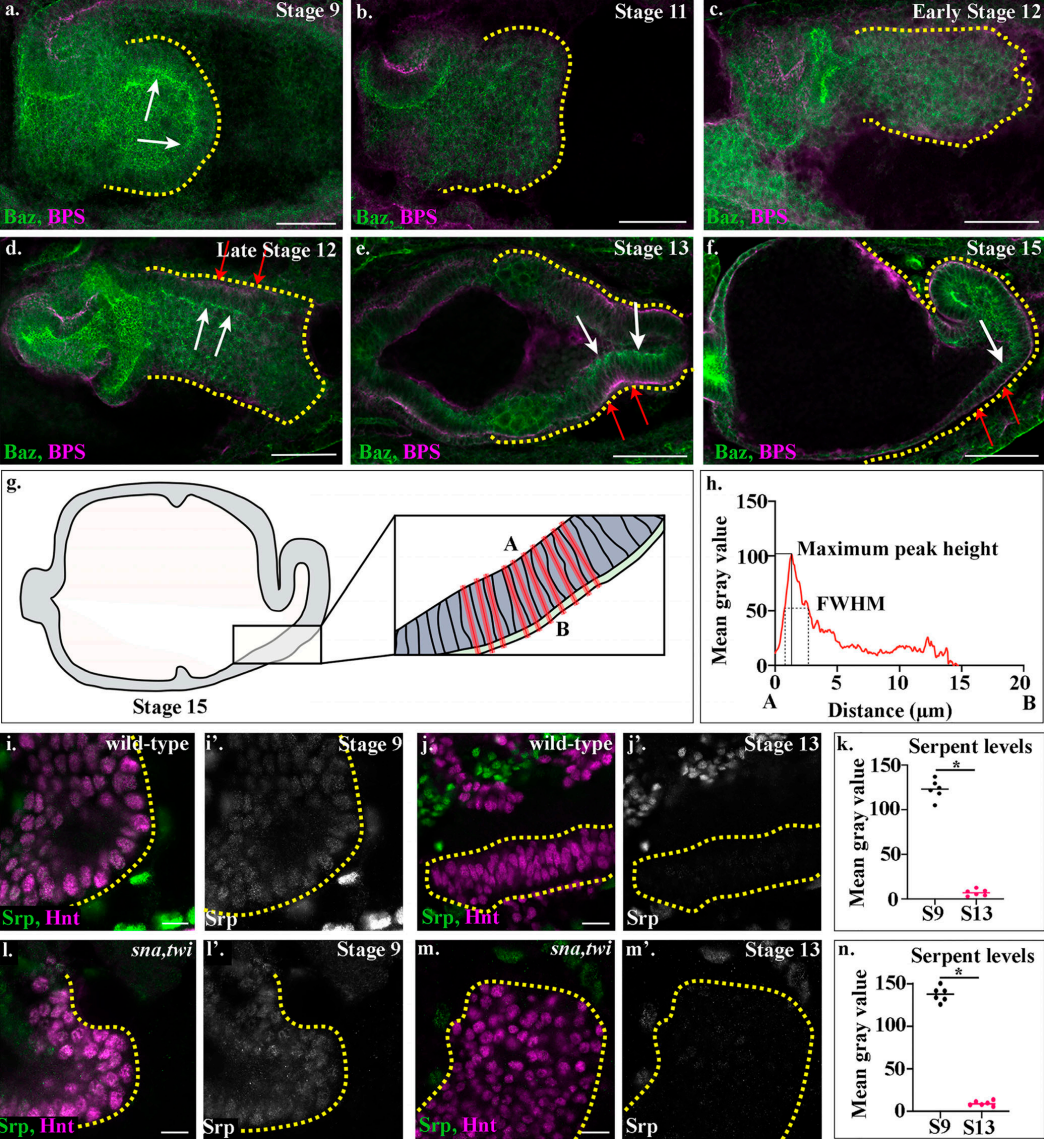
**Characterization of wild-type midgut MET and of Srp down-regulation in wild-type and *sna,twi* embryos.(a–f, i, j, l, and m)** Wild-type (a–f, i, and j) and *sna,twi* (l and m) embryos stained for Baz (green, a–f) and βPS (magenta, a–f) or Srp (green, i–m) and Hnt (magenta, i–m). White arrows (a and d–f) point to Baz localized apically in midgut cells, and red arrows (d–f) to βPS localized basally. Yellow dotted lines outline the posterior midgut. **(g)** Schematic depicting where measurements of fluorescence intensity were performed. The length of each cell within the ventral posterior region of the midgut was measured (boxed region) by drawing lines in the plane of the cell, from 1 µm above the apical membrane (A) of midgut cells to 1 µm below the basal surface (B) of the visceral mesoderm in a single z-slice. **(h)** FWHM was measured by calculating the width of the peak at half maximum peak height per embryo. Peak height was measured by finding the maximum value of peaks within specific ranges of the cell length (0–10 µm for apical peaks and 10–20 µm for basal peaks). **(k and n)** Dot plots of Srp levels in wild-type (k) or *sna,twi* (n) embryos. The Srp level per embryo at each stage is calculated as the mean gray value of 30 individually measured nuclei, and each dot represents one embryo. Each *n* represents the average mean gray value of 30 individually measured nuclei in one embryo. *n* = 6 per time point, per condition. *, P < 0.001 by unpaired two-tailed *t* test.

To test the relationship between EMT-TF down-regulation and MET, we previously followed these processes in *Drosophila* embryonic midgut formation. The midgut originates from two groups of endodermal cells at each pole of the blastoderm embryo. During gastrulation, cells that will form the anterior and posterior midgut undergo EMT, converting to unpolarized masses of mesenchymal cells, which migrate through the embryo (Fig. S1, a–c; Campbell et al., 2011). These cells later undergo MET to give rise to a large portion of the intestinal tract (Fig. S1, d–f; Campbell et al., 2011; Campos-Ortega and Hartenstein, 1985; Reuter, 1994; Skaer, 1993; Tepass and Hartenstein, 1994), showing remarkable parallels with cell changes seen during vertebrate endoderm morphogenesis (Nowotschin et al., 2019).

**Figure 1.**
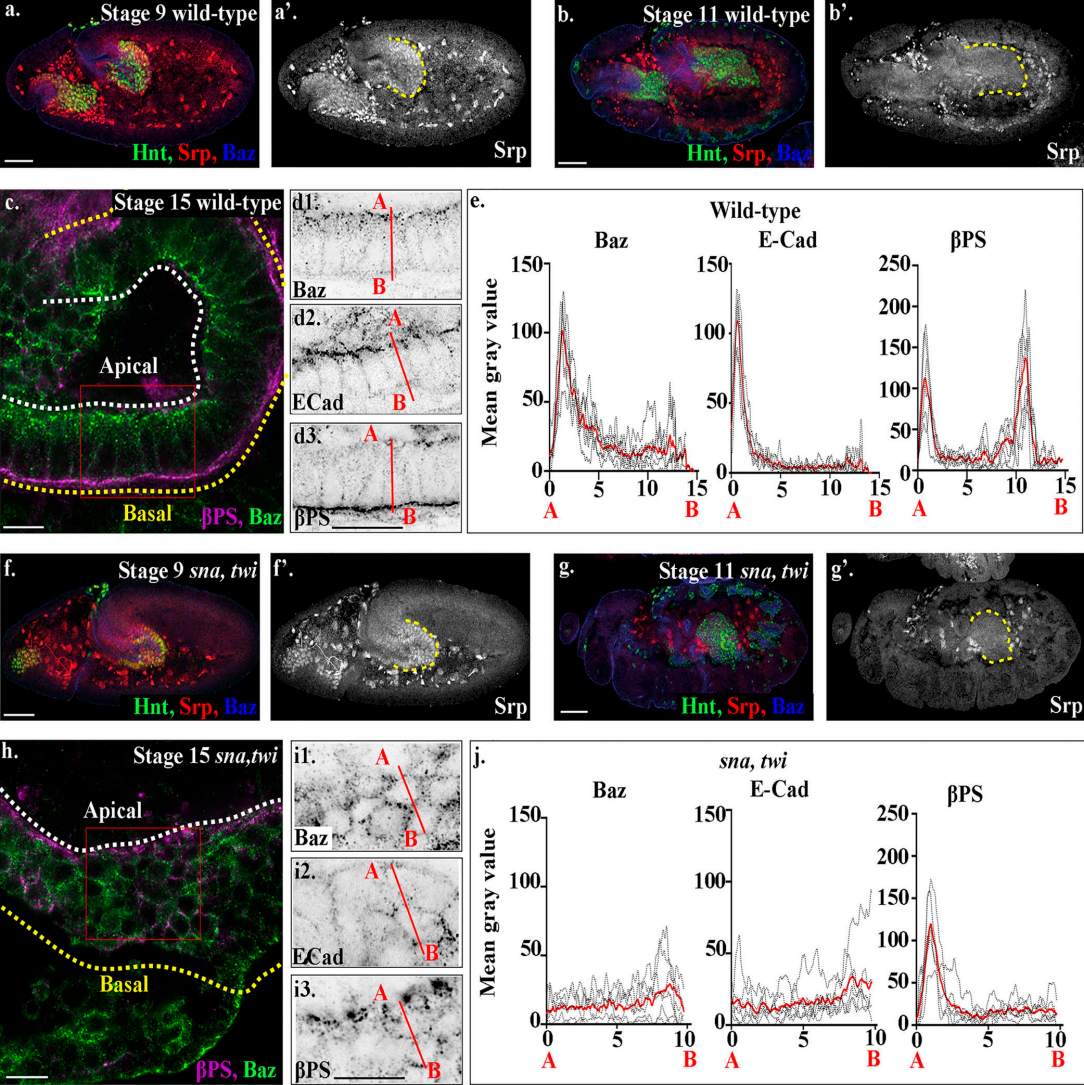
**Serpent down-regulation is not sufficient for midgut MET. (a–d)** Wild-type embryos before EMT (stage 9; a), during migration (stage 11; b), and after MET (stage 15; c and d). **(a and b)** Serpent is expressed in midgut cells before EMT (red; a) and starts being down-regulated after EMT, as the cells initiate migration (red; b). **(c and d)** By stage 15, midgut cells have formed a monolayer of columnar-shaped cells, with Baz (d1) and E-Cad (d2) localized to the apical side of the cells and βPS strongly localized to both the basal and apical domains (d3). **(e)** Plots of the average fluorescence intensity (represented as mean gray value) of Baz, E-Cad, and βPS in stage 15 midgut cells measured along the apical (A) to basal (B) axis of a cell (represented in d by red line). For every such plot of average fluorescence shown in this paper, each dotted black line represents the average of a minimum of 10 cells per embryo. The solid red line represents overall the average of six embryos. See Materials and methods for how quantifications were performed. Sharp peaks of Baz and E-Cad are seen at the apical side of the cell, with peaks for βPS integrins seen both apically and basally, with the basal peak of βPS slightly higher than the apical peak. **(f–i)** *sna,twi* mutants before EMT (stage 9; f), during migration (stage 11; g), and at stage 15 (h and i). In s*na,twi* double mutant embryos, Srp is expressed before EMT (f) and down-regulated by the start of germband retraction (stage 11; g), as in wild-type (a and b). **(h)** Stage 15 midgut cells in *sna, twi* mutants are rounded and multilayered. **(i)** Baz (i1), E-Cad (i2), and βPS (i3) do not localize as in wild-type (compare *sna,twi*, h and i, with wild-type, c and d). **(j)** Plots of the average fluorescence intensity across *sna,twi* mutant midgut cells show that peaks of Baz, E-Cad are lost. While the basal peak of βPS is completely absent, the apical peak of βPS is still there. *n* = 6 embryos per condition (minimum 10 cells per embryo). The posterior midgut in (a, b, f, and g) is demarcated by dashed yellow lines, and Hindsight (Hnt, green) labels midgut cells. White dashed lines in a and h indicate the apical side of the midgut, and yellow lines, the basal. Red boxes in c and h depict the area of the midgut epithelium shown in d and i. Confocal images are oriented with the anterior to the left and posterior to the right. Scale bars, 10 µm (c, d, h, and i) and 50 µm (a, b, f, and g).

We previously showed that EMT in the *Drosophila* posterior midgut is driven by activation of Serpent, the orthologue of human GATA TFs 4/6. Serpent is down-regulated in these cells shortly after EMT has taken place (Fig. 1, a and b; Campbell et al., 2011; see Fig. S1 k for quantification of Serpent levels before and after migration). When we forced sustained Serpent expression in midgut cells, preventing timely down-regulation, midgut cells failed to undergo MET and never regained epithelial characteristics (Campbell et al., 2011). Thus, as seen in many other systems (Ocaña et al., 2012; Tran et al., 2014; Tsai et al., 2012), Serpent down-regulation is necessary for MET to occur.

### Midgut cells progressively establish apicobasal polarity and epithelial organization after contact with the visceral mesoderm

**Figure S2.**
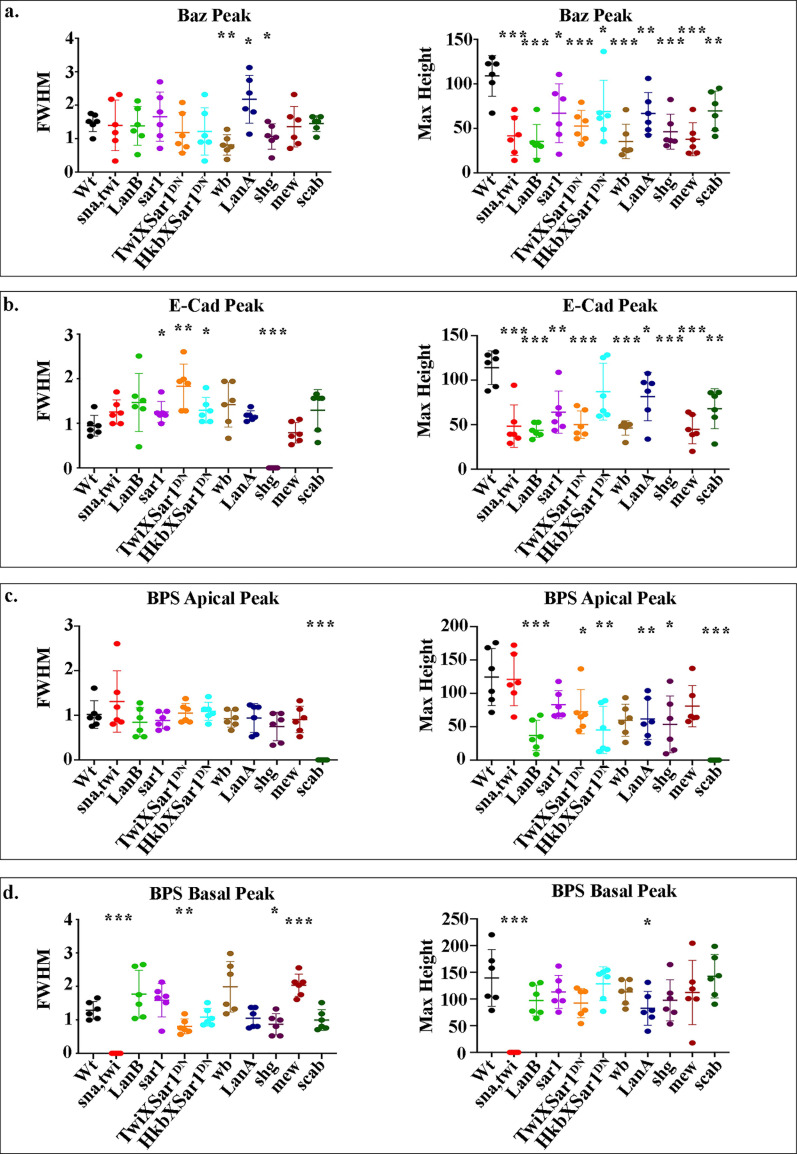
**FWHM and amplitude (maximum height) of fluorescence intensity peaks.(a–d) **Measurements of the FWHM determine the spread away from the peak, and the amplitude of fluorescence intensity peaks indicates relative protein concentrations. Data are displayed as scatter plots: each dot represents the average of a minimum of 10 cells measured in one embryo, with mean and SD indicated by horizontal lines. Each *n* represents the average of 10 cells measured in one embryo. *n* = 6 per condition. Statistical analyses were performed comparing mutants to wild type (Wt) for each peak using unpaired two-tailed *t* test. ***, P ≤ 0.001; **, P ≤ 0.01; *, P ≤ 0.05; no asterisk, nonsignificant.

After undergoing EMT, midgut cells migrate across the visceral mesoderm. Indeed, direct contact between the visceral mesoderm and endoderm is known to be required not just for their migration, but also for midgut cells to undergo MET and form an epithelium (Reuter et al., 1993; Tepass and Hartenstein, 1994). To characterize and determine the timing of midgut MET, we examined midgut cell morphology and the expression/localization of markers of apicobasal polarity at distinct stages of development. Midgut cells lose their columnar shape during EMT, as well as tight localization of apicobasally polarized proteins such as Crumbs, E-cadherin (E-Cad), and the Par3 homologue Bazooka (Baz; Fig. S1, a and b; Campbell et al., 2011). While apical proteins such as Crumbs and Stardust are transcriptionally repressed during midgut EMT, Baz and E-Cad remain highly expressed, although the proteins become delocalized (Campbell and Casanova, 2015; Campbell et al., 2011). Midgut cells stay rounded as they contact the visceral mesoderm and initiate migration and show no indication of apicobasal polarity (Fig. S1 c). Midway through their migration, midgut cells start to adopt an elongated columnar shape and to localize Baz apically and the β-position–specific integrin subunit (βPS) basally (Fig. S1 d). After midgut cells have finished migration, their columnar organization and localization of Baz and βPS are more pronounced (Fig. S1, e and f). To examine this in more detail, we looked at the midgut in stage 15 embryos using high-resolution confocal imaging (63× lens combined with Airyscan; Carl Zeiss Microscopy). We found that both Baz and E-Cad are localized to the apical membrane (Fig. 1, c and d). In contrast, βPS is tightly restricted to the basal side of the cells, although lower levels can also be seen apically (Fig. 1, c and d). To understand this further and form a foundation for comparing mutant phenotypes, we plotted the average fluorescence intensity (represented as mean gray value) of Baz, E-Cad, and βPS along a line drawn through the center of midgut cells, from the apical to the basal surface (Fig. 1, d and e; see Materials and methods and Fig. S1 g for how plots were constructed). We measured the amplitude of fluorescence intensity peaks as readouts for relative protein concentrations and the full width at half maximum (FWHM) as an indicator of spread away from the peak (Figs. S1 h and S2). These plots show clear apical peaks for Baz and E-Cad, with both proteins almost completely absent in other parts of the cell. Interestingly, this analysis reveals βPS to have a peak at both the apical surface, where the midgut cells border the amnioserosa, and at the basal surface, where the cells meet the underlying visceral mesoderm, with the basal peak slightly higher than the apical (Figs. 1 e and S2).

### Down-regulation of the EMT-TF Serpent is not sufficient for MET

Studies from metastasis models suggest that contact between circulating tumor cells and the metastatic niche trigger EMT-TF down-regulation and MET (Del Pozo Martin et al., 2015; Gao et al., 2012). We therefore hypothesized that as Serpent down-regulation appears to occur concurrently with the first signs of midgut MET, contact with the visceral mesoderm leads to a down-regulation of Serpent in midgut cells, in turn triggering MET. To test this hypothesis, we followed Serpent expression dynamics in midgut cells, in embryos mutant for both *snail* (*sna*) and *twist* (*twi*), i.e., in a background with complete loss of mesoderm derivatives (Reuter et al., 1993; Tepass and Hartenstein, 1994). In embryos lacking mesoderm, Serpent is expressed very strongly in the posterior midgut before EMT and subsequently down-regulated in these cells as in wild type (compare Fig. 1, f and g, with wild-type Fig. 1, a and b; see Fig. S1 n for quantification of Serpent levels before and after migration in *sna,twi* mutants). However, despite down-regulating Serpent, posterior midgut cells do not undergo MET, instead remaining as a multilayered cord of rounded cells, which fail to localize Baz, E-Cad, and βPS as in wild type (Fig. 1, h–j; and Fig. S2). Interestingly, the apical peak of βPS is still apparent, despite the disorganization of the midgut cells, suggesting that βPS localizes in midgut cells at the endoderm/amnioserosa border independently of midgut MET (Fig. 1, h–j; and Fig. S2). Taken together, these findings demonstrate that in vivo, where cells can be followed throughout migration and MET, down-regulation of an EMT-TF is not sufficient to drive MET. Furthermore, EMT-TF down-regulation and MET are separable events. Finally, they suggest that MET requires additional inputs from neighboring tissues to drive the repolarization and reepithelialization of cells.

### Interactions with laminins are required for correct midgut migration and MET

**Figure 2.**
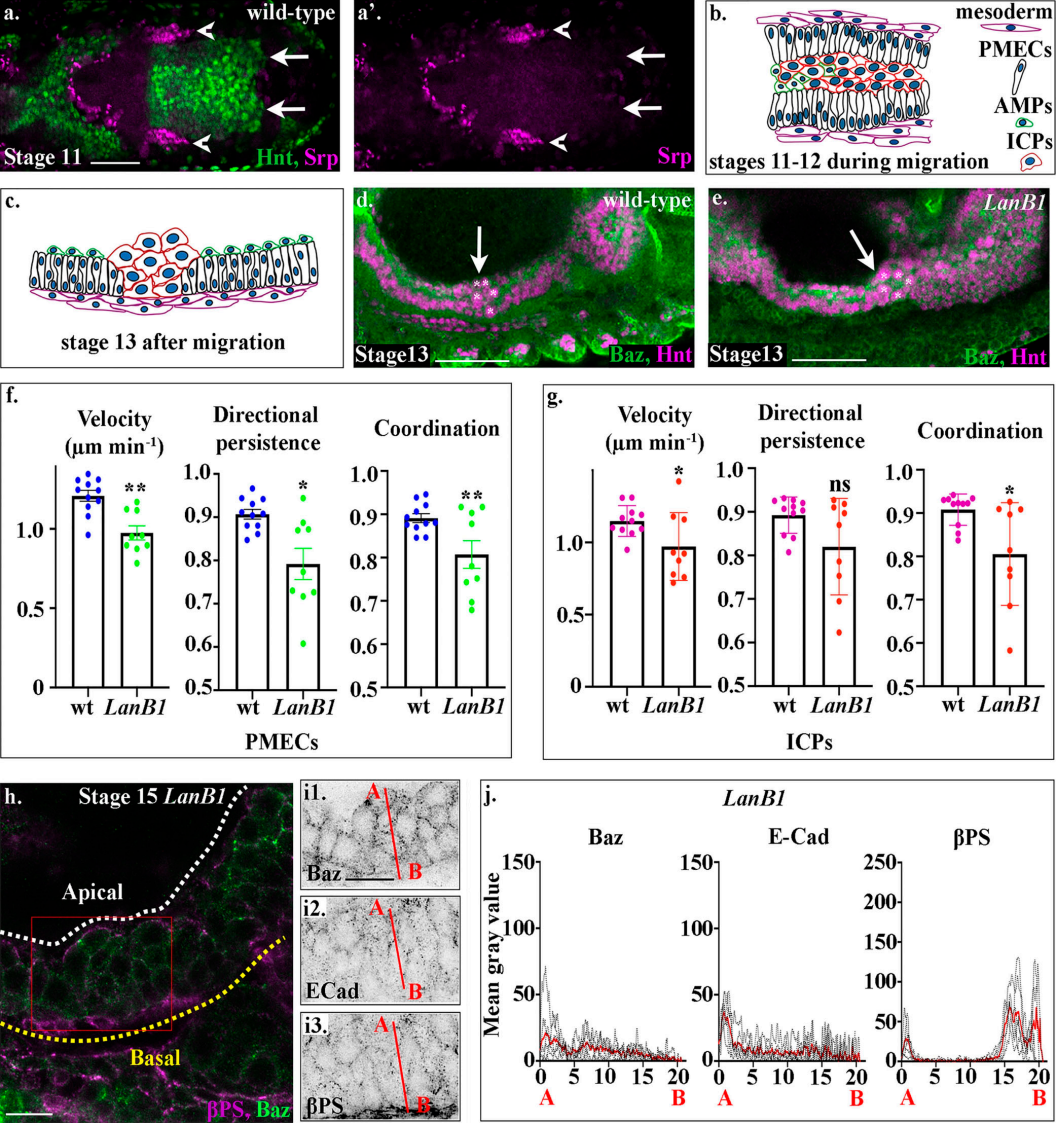
**Laminins are required for midgut migration and MET.(a)** A dorsal cross-section of a stage 12 wild-type embryo shows that at this stage, hemocytes (red) have not yet migrated to surround the midgut (compare the tip of the migrating hemocytes, arrowheads, with the leading edge of the posterior midgut, arrows). **(b)** Midgut cells migrate in a highly coordinated fashion, with PMECs on the outside, adjacent to the mesoderm, and the ICPs and AMPs on the inside of a PMEC “sandwich.” **(c)** After migration, midgut cells take up highly stereotypical positions, ICPs cluster between the anterior and posterior portions of the midgut, the PMECs form a monolayer contacting the visceral muscle, and the AMPs sit on the apical side of the PMECs. **(d)**. In wild-type embryos, the anterior midgut and posterior midgut have met and fused by stage 13, and the ICPs (asterisks) sit in the middle (d, arrow). **(e)** In *LanB1* mutants, the ICPs (asterisks) fail to migrate to the middle and are situated more posteriorly (e, arrow). **(f and g)** Velocity, directional persistence, and coordination values calculated from videos of wild-type (*n* = 11) and *LanB1* mutant (*n* = 9) embryos. *n* represents the average of a minimum of 15 cells in one embryo (shown as a dot). Data are presented as mean ± SEM. *, P ≤ 0.05; **, P ≤ 0.01 by unpaired two-tailed *t* test (see Table S1 for raw data). **(h)** Stage 15 midgut cells in *LanB1* mutants are multilayered and rounded. **(i)** Baz (i1), E-Cad (i2), and βPS (i3) do not show any polarized localization within stage 15 midgut cells. βPS appears diffuse in the visceral mesoderm (i3). **(j)** Plots of the average fluorescence intensity (represented as mean gray value) of Baz, E-Cad, and βPS in stage 15 *LanB1 *mutant midgut cells measured along the apical (A) to basal (B) axis of a cell (represented in b by red line). *n* = 6 embryos per condition (minimum 10 cells per embryo). The sharp peaks of Baz and E-Cad seen in wild-type cells are lost. While apical and basal peaks of βPS are absent, there is a low broad peak of βPS that spans the underlying mesoderm layer, indicating that βPS is down-regulated in midgut cells and delocalized in the mesoderm. White dashed lines in h indicate the apical side of the midgut, and yellow lines, the basal. Red box in h depicts the area of the midgut epithelium shown in i. Confocal images are oriented with the anterior to the left and posterior to the right. Scale bars, 50 µm (a, d, and e) and 10 µm (h and i).

While upstream cues that trigger MET in the midgut are not known, previous studies have implicated integrin receptors in mediating the repolarization of the posterior midgut (Devenport and Brown, 2004; Martin-Bermudo et al., 1999; Tepass and Hartenstein, 1994). As integrins mediate key contacts between the cell and ECM, this suggests that interactions with ECM components from neighboring tissues may be required for MET. However, midgut migration occurs very early in development, at a stage when many of the major components of the ECM may not yet be present in the embryo. Recent studies have revealed that *Drosophila* hemocytes are responsible for depositing a subset of ECM components as they migrate from the head of the early embryo and disperse throughout the body (Matsubayashi et al., 2017). While hemocytes have been reported to initiate migration during early stages of embryogenesis (Siekhaus et al., 2010), it was not clear whether they actually reached the midgut by the time migration started, and therefore whether they act in time to contribute ECM components for midgut migration. We investigated this in detail by imaging hemocytes and midgut migration simultaneously. We found that at the time of midgut migration, the hemocytes have not yet migrated from the anterior region of the embryo (Fig. 2 a) and do not surround the posterior midgut until after migration has completed and MET has occurred (Video 1). This finding rules out hemocytes as a source of substrate for midgut migration. As hemocytes are the only source of Collagen IV (Col IV) and Perlecan (Perl) at this stage of development (Matsubayashi et al., 2017), it also suggests that the integrin-mediated midgut migration and MET must be independent of interactions with the ECM components Col IV and Perl.

**Video 1.** **Time-lapse video of hemocyte and midgut migration.** The video begins at the initiation of germband retraction, hemocytes are visualized by serpentHemoGal4 (srp-Gal4, magenta), and the nuclei of midgut cells are visualized by UAS-StingerGFP (green) under the control of HkbGal4. Frames are taken every 2 min, and a maximum projection of three z-slices of 1.5-µm thickness is shown. Playback speed, 7 frames/s.

Another major component of the ECM are the laminins, which have previously been shown to play a key role in the migration of several embryonic cell populations in different species (Brown, 2011; Sánchez-Sánchez et al., 2017). Laminin mRNAs are expressed far earlier in *Drosophila* development than Col IV and Perl (Matsubayashi et al., 2017; Wolfstetter and Holz, 2012). In *Drosophila,* four *Laminin* genes have been identified that encode one β chain (*lamininB1*; *LanB1*), one γ chain (*lamininB2*;* LanB2*), and two α chains: an α1,2 homologue, encoded by *wb*, and an α3,5 laminin, *lamininA (LanA).* Together these chains form two heterotrimers that differ only in their α subunit: lamininW, which contains Wb, and lamininA, which contains LanA. The requirement for laminins during *Drosophila* morphogenesis has previously been investigated by looking at mutants lacking *LanB1* and/or *LanB2*, thus removing both heterotrimers. Loss of LanB1 has been noted to produce delays in midgut cell migration (Urbano et al., 2009), while defects in both midgut migration and repolarization have been noted in *LanB2* mutants (Wolfstetter and Holz, 2012), supporting a role for laminins in mediating some or all aspects of midgut migration and MET.

Midgut cells migrate in a stereotypical and coordinated manner as a heterogenous population of epithelial-like and mesenchymal cells (Campbell and Casanova, 2015). During endodermal EMT, the cells that will form the midgut are subdivided into three populations of cells: the principle midgut epithelial cells (PMECs), the cells that undergo MET and form the embryonic and larval epithelial midgut (Campbell and Casanova, 2015; Tepass and Hartenstein, 1995), and the interstitial cell precursors (ICPs) and adult midgut precursors (AMPs), which remain mesenchymal throughout most stages of embryogenesis (Campbell and Casanova, 2015; Tepass and Hartenstein, 1995). Migration of midgut cells is highly coordinated, with PMECs migrating on the outside of the cluster, in direct contact with the neighboring mesoderm, and the mesenchymal ICPs and AMPs sandwiched in the middle (Fig. 2 b; ICPs can be distinguished from PMECs and AMPs by their large nuclei; Campbell and Casanova, 2015). At the end of migration, the cells take up highly stereotypical positions within the embryo: the ICPs cluster in the middle, between the anterior and posterior portions of the midgut; the PMECs form an epithelial monolayer adjacent to the visceral muscle; and the AMPs sit on the apical side of the PMECs bordering the yolk (Fig. 2, c and d).

**Figure S3.**
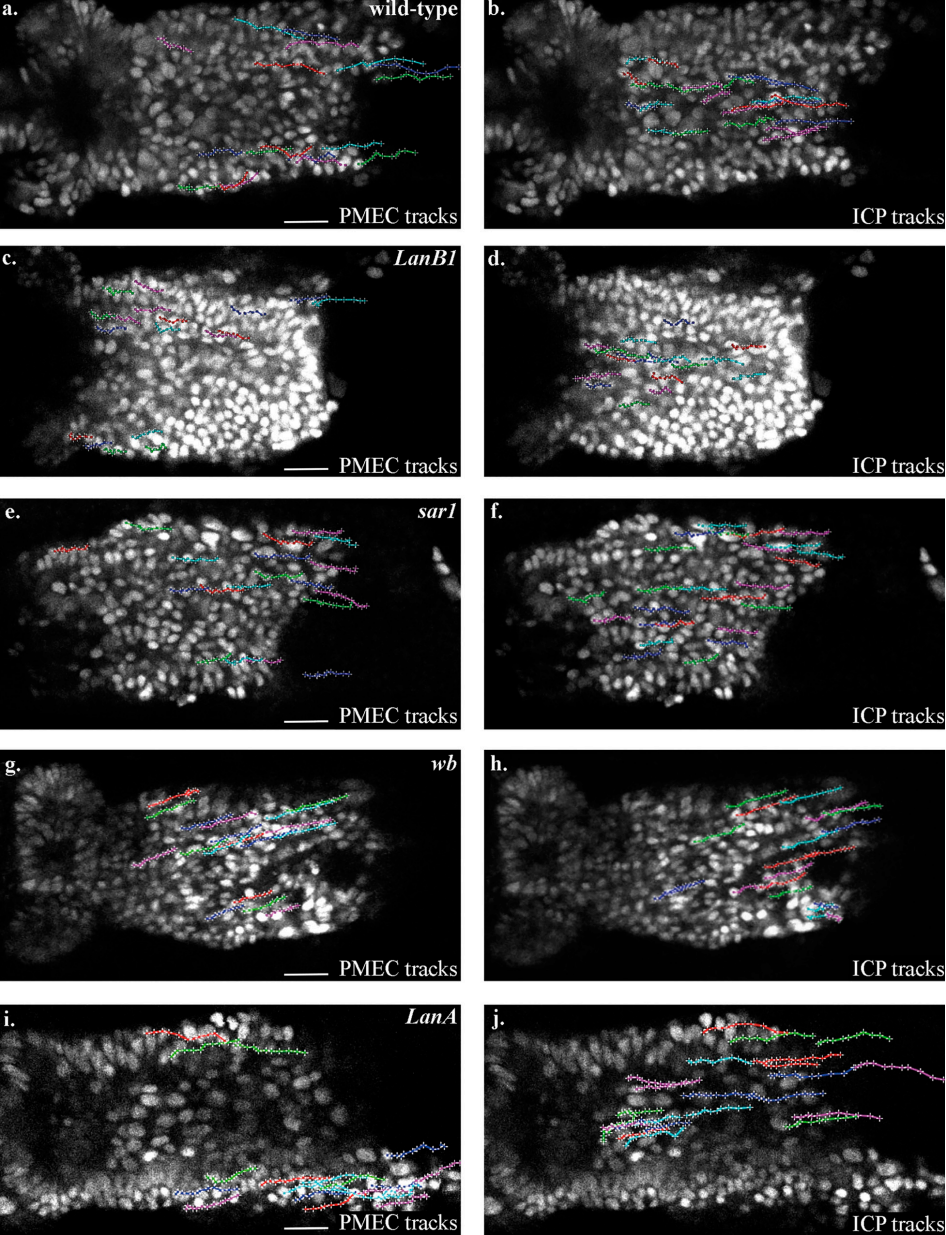
**Representative tracks of paths taken by PMECs and ICPs in each genotype where cells were tracked in this work.** PMECs and ICPs are identified by their nuclear diameter (PMECs, <3.5 µm; ICPs, >5.5 µm). To aid comparison, tracks are arbitrarily labeled in red, blue, and green. **(a and b)** PMEC (a) and ICP (b) tracks in wild-type embryos. **(c and d)** PMEC (c) and ICP (d) tracks in LanB1 mutant embryos. **(e and f) **PMEC (e) and ICP (f) tracks in sar1 mutant embryos. **(g and h)** PMEC (g) and ICP (h) tracks in wb mutant embryos. **(i and j)** PMEC (i) and ICP (j) tracks in LanA mutant embryos. Scale bars, 20 µm.

When examining the midgut in embryos lacking *LanB1*, we noticed that mesenchymal ICPs fail to move to the center point between the anterior and posterior midgut rudiments and are located more posteriorly than in wild type (Fig. 2 e). To pinpoint when and where laminins are required during midgut migration, we tracked subsets of PMEC and ICP cell nuclei using a combination of custom-built ImageJ macros that allow the automated 3D tracking and manual validation of each track (as in Campbell and Casanova [2015] and Tosi and Campbell [2019]). From these tracks, we calculated the average velocity and directional persistence of individual cells, as well as coordination of migration between neighboring cells. In wild-type embryos, both PMECs and ICPs migrate highly directionally, with a high degree of coordination and a velocity of 1.2 µm min^−1^ at 25°C (Fig. 2, f and g; Fig. S3; and Video 2; see Table S1 for raw data). In *LanB1* mutant embryos, we found that the migration of both PMECs and ICPs is significantly altered: they migrate slower than in wild type and display a lower degree of coordination (Fig. 1, f and g; Fig. S3; and Video 3). While the directional persistence of the PMECs is reduced, the lower average persistence in the ICP cells is not significant (Fig. 2, f and g). This indicates that the delay in midgut migration previously observed in *LanB1* mutant embryos (Urbano et al., 2009) is due to a slower, less directional, and less coordinated migration of the epithelial-like PMECs, as well as a slower velocity and coordination in the mesenchymal ICPs. The maintenance of directional persistence in ICP cells, despite a lower degree of coordination, may reflect spatial constraints imposed by surrounding PMECs.

**Video 2.** **An example of a time-lapse video that was used for the tracking of migrating PMG cells in wild-type embryos.** PMG cell nuclei were labeled by StingerGFP. The video begins at the initiation of germband retraction, frames are taken every 2 min, and a single z-slice is shown. Videos are tracked in 4D over multiple z-slices of 1.5-µm thickness. Playback speed, 3 frames/s.

**Video 3.** **An example of a time-lapse video that was used for the tracking of migrating PMG cells in *LanB1* mutant embryos.** PMG cell nuclei were labeled by StingerGFP. The video begins at the initiation of germband retraction, frames are taken every 2 min, and a single z-slice is shown. Videos are tracked in 4D over multiple z-slices of 1.5-µm thickness. Playback speed, 3 frames/s.

We next examined the midgut cells of *LanB1* mutants to see if they undergo MET, as defects in the formation of the midgut epithelium have been observed in *LanB2* mutants (Wolfstetter and Holz, 2012). In stage 15 *LanB1* mutants, midgut cells are rounded and multilayered. High-resolution confocal imaging of Baz and E-Cad reveals that these proteins are not localized and appear diffuse throughout the midgut cells (Fig. 2, h and i). Accordingly, plots of the fluorescence intensities from the apical to basal sides of the midgut show that the localized peaks of these proteins are lost (Figs. 2 j and S2). Confocal images show a loss of βPS localized to the endoderm/mesoderm interface and diffuse βPS staining throughout the visceral mesoderm (Fig. 2, h and i). This appears in the quantification as a basal plateau with a FWHM of nearly 2 µm instead of the 1.3 µm seen in wild-type cells (Figs. 2 j and S2). Taken together, these data indicate that either one or both laminin heterotrimers are required for midgut cells to form a monolayer of regular columnar-shaped cells, and for the establishment of apicobasal polarity.

### The secretion of laminins from both the midgut and surrounding mesoderm contribute to midgut migration and MET

**Figure 3.**
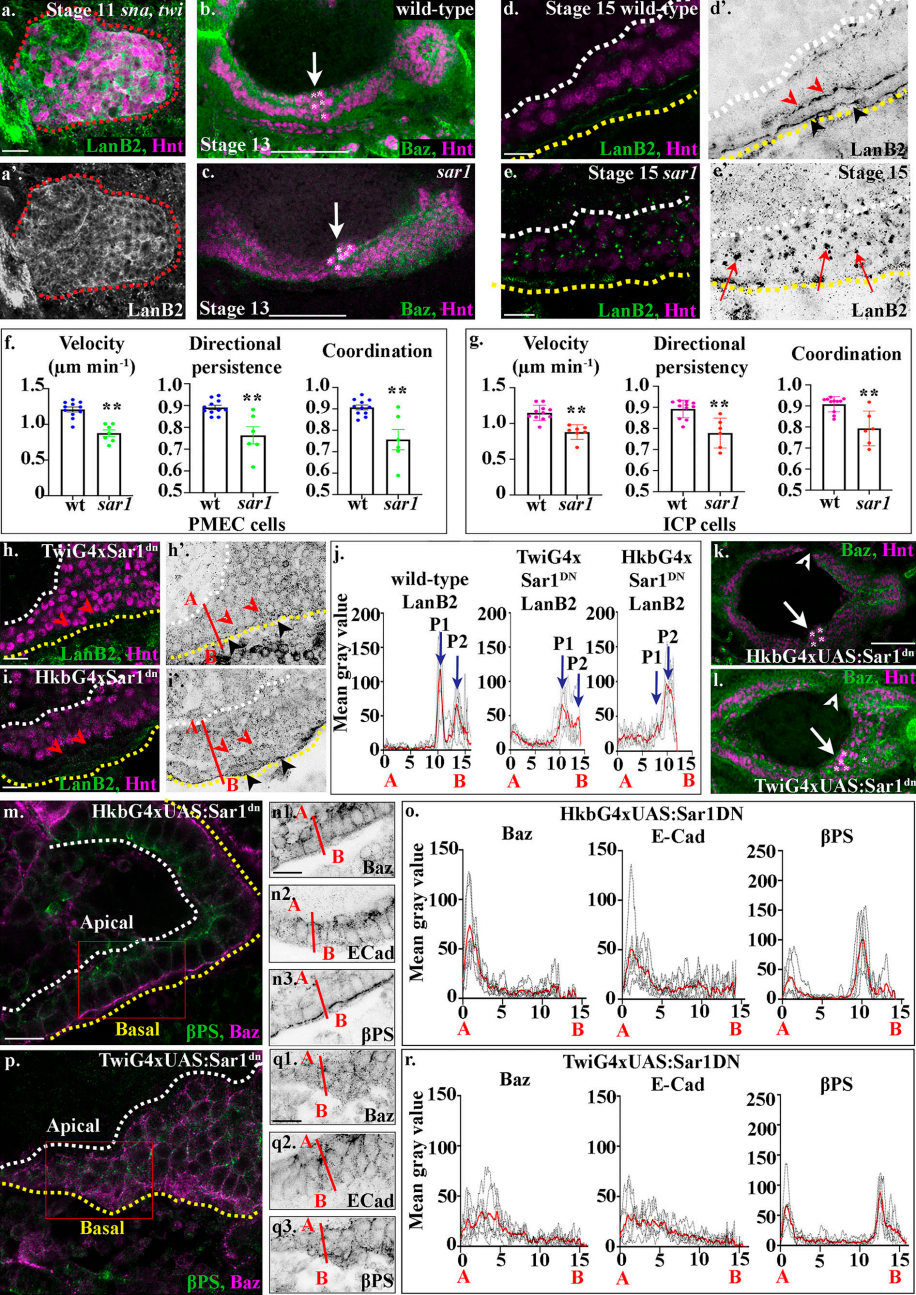
**Secretion of laminins via the CopII pathway, from both the mesoderm and the midgut cells themselves, is required for their correct migration and MET**. **(a)** Laminins (green) are strongly expressed in the posterior midgut cells in *sna,twi* mutant embryos. The posterior midgut is demarcated by dashed red lines. **(b and c)** In stage 13 *sar1* zygotic mutants, the ICP cells (asterisks) are found more posteriorly than in wild-type (compare c with wild-type b; the same wild-type was used as in Fig. 2 d for comparison). **(d)** In the wild-type stage 15 midgut epithelium, two layers of laminin are found on the basal surface, one at the endoderm/mesoderm border (d′, red arrowheads) and the second on the outer surface of the mesoderm (d′, black arrowheads). Additionally, a lower level of LanB2 at the apical surface of midgut cells can be seen (d′, by white dotted line). **(e)** In stage 15 *sar1* mutants, large intracellular punctae of laminins can be found (e, red arrows), indicating defects in laminin secretion (*n* = 40 embryos at stage 15; all embryos examined had multiple intracellular punctae of LanB2; representative image shown in e). **(f and g)** Velocity, directional persistence, and coordination values calculated from videos of wild-type (*n* = 11) and *sar1* (*n* = 6) mutant embryos. *n* represents the average of a minimum of 15 cells in one embryo (shown as a dot). Data are presented as mean ± SEM. **, P ≤ 0.01 by unpaired two-tailed *t* test (see Table S1 for raw data). **(h)** TwiGal4 driving Sar1^DN^ in the mesoderm leads to a loss of LanB2 staining at the endoderm/mesoderm interface (red arrowheads). Low levels of LanB2 staining can be seen throughout the mass of rounded midgut cells (black arrowheads point to the basal side of the visceral mesoderm). **(i)** HkbGal4 driving Sar1^DN^ in the endoderm leads to a loss of LanB2 staining at the endoderm/mesoderm interface (red arrowheads), while LanB2 can still be seen on the opposite side of the mesoderm (black arrowheads). **(j)** Plots of the average fluorescence intensity (represented as mean gray value) of LanB2 in stage 15 midgut cells measured along the apical (A) to basal (B) axis of a cell (represented in b by red line). Each *n* represents the average of 10 cells measured in one embryo (black dotted lines). *n* = 6 per condition; the mean for each condition is plotted in red. In wild-type cells there is a peak of LanB2 at the endoderm mesoderm border (P1) and on the outer surface of the mesoderm (P2). In TwiG4xSar1^DN^, LanB2 levels are reduced, P1 appears as a broad diffuse peak, and P2 is either absent or greatly reduced (see Fig. S5 for FWMH). In HkbG4xSar1^DN^, the endoderm/mesoderm peak, P1, is either absent or greatly reduced, while more diffuse levels of LanB2 can still be seen on the opposite side of the visceral mesoderm (P2 FWMH increases from 1 µm in wild type to 1.8 µm; see Fig. S5). **(k and l)** Stage 13 embryos expressing Sar1^DN^ in either the endoderm using HkbG4 (k) or mesoderm using TwiG4 (l). In both conditions, midgut migration is delayed; gaps are found between the anterior and posterior midgut rudiments (arrowheads) which have normally fused by stage 13; and the ICPs (asterisks) are found more posteriorly than in wild type (arrows). *n* = 40 embryos per condition; all embryos show gaps and/or mispositioned ICPs; representative images shown. **(m–o)** When the CopII pathway is blocked in midgut cells, they form a disorganized-looking monolayer of columnar or wedge-shaped cells, with βPS integrins localized to the apical and basal sides of the cells (n3), although quantification of levels reveals the peaks of βPS to be half that in wild type (o). In contrast, Baz (n1) and E-Cad (n2) are no longer restricted to the apical side of the cells (m–o) and are found in lower levels throughout the lateral membranes (m–o). **(p–r)** When Sar1^DN^ is expressed in the mesoderm, midgut cells fail to form a monolayered epithelium, remaining rounded and multilayered (p), and Baz (q1) and E-Cad (q2) do not show any polarized localization within the midgut cells. βPS (q3) levels are very reduced, and basal βPS staining appears lost in midgut cells and diffuse throughout the underlying visceral mesoderm (p, q3, and broad basal peak in r). o and r show plots of the average fluorescence intensity (represented as mean gray value) of Baz, E-Cad, and βPS in stage 15 midgut cells measured along the apical (A) to basal (B) axis of a cell (represented in n and q by red line). Each *n* represents the average of 10 cells measured in one embryo (black dotted lines). *n* = 6 per condition; the mean for each condition is plotted in red. White dashed lines in d, e, h, i, m, and p indicate the apical side of the midgut, and yellow lines, the basal. Red boxes in m and p depict the area of the midgut epithelium shown in n and q. Confocal images are oriented with the anterior to the left and posterior to the right. Scale bars, 20 µm (a), 50 µm (b, c, k, and l), and 10 µm (d, e, h, i, m, n, p, and q).

The requirement for the mesoderm and laminins for midgut migration and MET presents a simple model whereby the mesoderm secretes laminins and midgut cells use this, either alone or together with other mesodermal cues, as a substrate for migration and repolarization. However, while the mesoderm is widely thought to be the main source of laminin secretion during early embryogenesis (Kusche-Gullberg et al., 1992; Matsubayashi et al., 2017; Montell and Goodman, 1989; Sánchez-Sánchez et al., 2017), RNAs for *LanB1* and *LanB2* have recently been detected in the midgut cells themselves and shown to be directly activated by Serpent (Töpfer et al., 2019). This raises the question of whether midgut cells are producing their own ECM for migration. Indeed, when we examined the midgut in embryos that completely lack mesoderm, high levels of LanB2 protein were clearly detected in the endoderm (Fig. 3 a).

**Figure S4.**
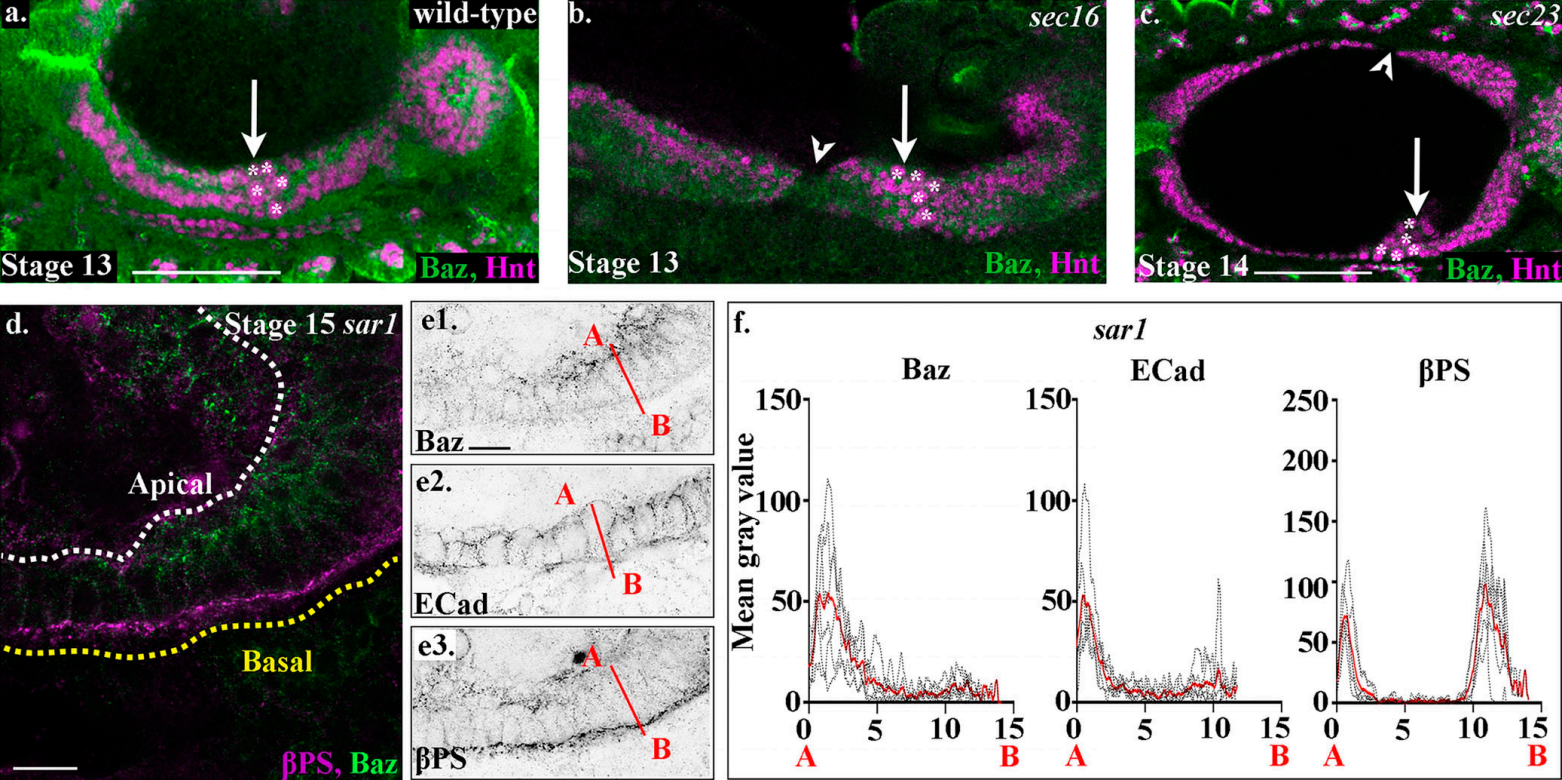
**Midgut migration and repolarization is perturbed in zygotic mutants for the CopII trafficking pathway.(a)** In wild-type embryos, the anterior midgut and posterior midgut have met and fused by stage 13, and the ICPs sit in the middle (a, arrow, asterisks). **(b)** In mutants for *sec16*, midgut migration is delayed, and gaps are seen between the anterior and posterior midgut rudiments (arrowhead). ICP migration is also perturbed, and they are found more posteriorly than in wild type (b, arrow, asterisks). **(c)** In *sec23* mutants, the midgut cells show defects similar to other CopII pathway zygotic mutants; there are gaps in the midgut (arrowhead), and the ICPs are found more posteriorly than in wild type (c, arrow, asterisks). **(d and e)** Midgut cells in stage 15 *sar1* mutant embryos. **(f)** Plots of the average fluorescence intensity (represented as mean gray value) of Baz, E-Cad, and βPS in stage 15 midgut cells mutant for *sar1* measured along the apical (A) to basal (B) axis of a cell (represented in e by red line). Each *n* represents the average of 10 cells measured in one embryo (black lines). *n* = 6 per condition; the mean for each condition is plotted in red. White dashed lines in a and b indicate the apical side of the midgut, and yellow lines, the basal. Scale bars, 50 µm (a–c) and 10 µm (d and e).

To investigate this further, we sought to find ways to block laminin secretion, which could then be used to investigate the tissue-specific contribution of laminin. Serendipitously, while testing different trafficking pathways for a putative role in midgut migration and MET, we noticed that several zygotic mutants for the CopII pathway show the same phenotype as embryos mutant for *LanB1*; ICP cells fail to move to the center point between the anterior and posterior midgut rudiments and are located more posteriorly than in wild type (Fig. 3, b and c; and Fig. S4, a–c). The CopII pathway regulates transport of proteins from the ER to the Golgi and has been shown to be required for the secretion of ECM molecules in a number of species (McCaughey and Stephens, 2018). Proteins involved in the CopII pathway such as Sar1, Sec16, and Sec23 have a strong maternal component. Zygotic mutants that lower the levels of these proteins can thus reveal cell processes that have an increased dependence on the trafficking of certain proteins through the pathway (Norum et al., 2010; Tsarouhas et al., 2007). In wild-type embryos, LanB2 is found to be enriched at the endoderm/mesoderm interface (Fig. 3 d, red arrowheads), as well as on the basal side of the visceral mesoderm (Fig. 3 d, black arrowheads). However, in *sar1* zygotic mutants, we find large intracellular punctae of LanB2, indicating defects in LanB2 secretion (Fig. 3 e, arrows). Accordingly, cell tracking of PMECs and ICPs in *sar1* mutant embryos reveals that, similar to *LanB1* mutant embryos, the migration of both cell types is significantly affected (Fig. 3, f and g; Fig. S3; and Video 4), and midgut cells in *sar1* mutants fail to repolarize and form an epithelial monolayer after migration (Fig. S4, d–f).

**Video 4.** **An example of a time-lapse video that was used for the tracking of migrating PMG cells in *sar1* mutant embryos.** PMG cell nuclei were labeled by StingerGFP. The video begins at the initiation of germband retraction, frames are taken every 2 min, and a single z-slice is shown. Videos are tracked in 4D over multiple z-slices of 1.5-µm thickness. Playback speed, 3 frames/s.

**Figure S5.**
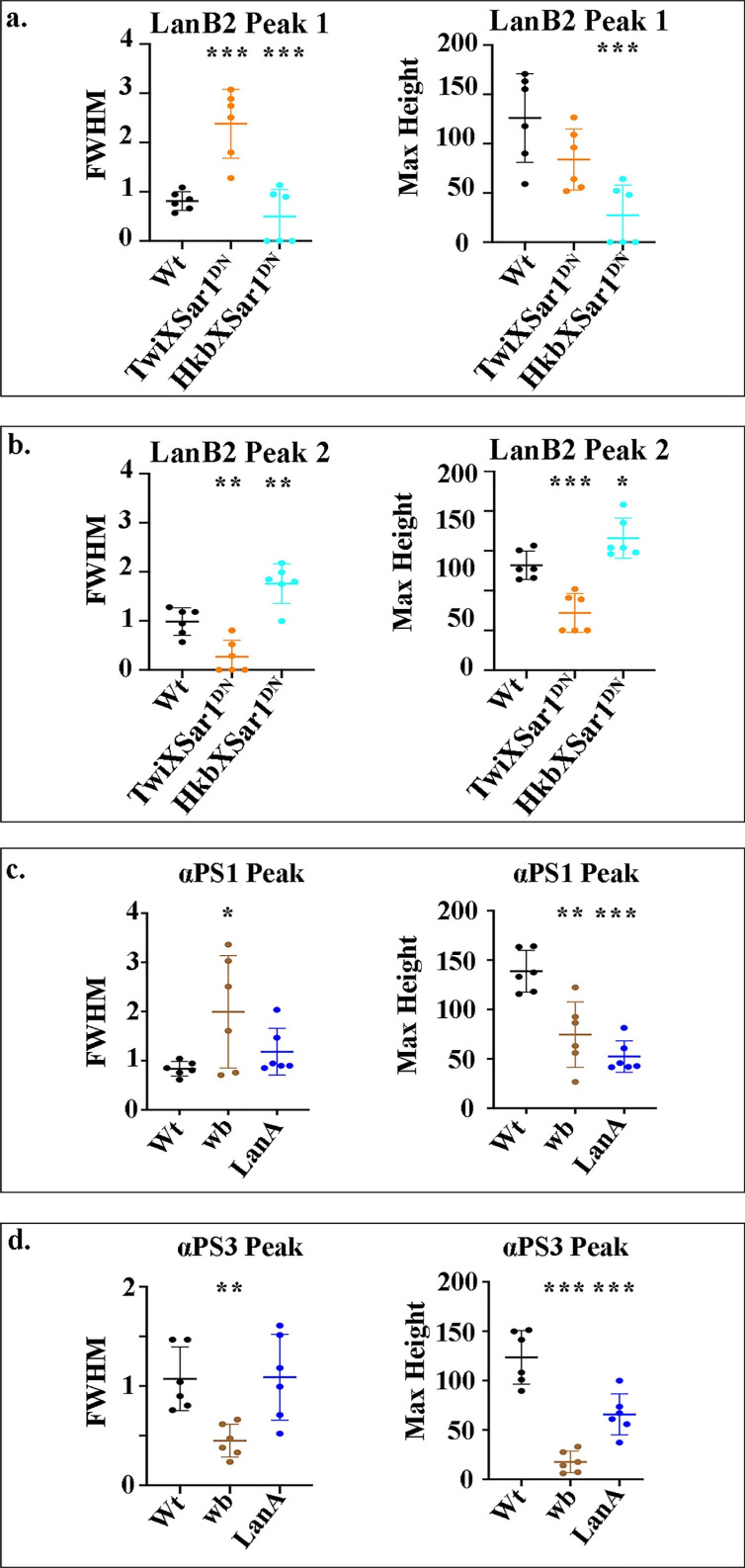
**FWHM and amplitude (maximum height) of fluorescent intensity peaks.(a–d) **Measurements of the FWHM determine the spread away from the peak, and the amplitude of fluorescent intensity peaks indicates relative protein concentrations. Data are displayed as scatter plots: each dot represents the average of a minimum of 10 cells measured in one embryo, with mean and SD from the mean indicated by horizontal lines. Each *n* represents the average of 10 cells measured in one embryo. *n* = 6 per condition. Statistical analyses were performed comparing mutants to the wild-type (Wt) for each peak using unpaired two-tailed *t* test. ***, P ≤ 0.001; **, P ≤ 0.01; *, P ≤ 0.05; no asterisks, nonsignificant.

As these data strongly suggested that Sar1 is required for laminin secretion, we next used a dominant-negative Sar1 construct (*sar1*^T38N^; Kuge et al., 1994; Tsarouhas et al., 2007), which can be expressed under the control of tissue-specific Gal4 drivers, to ask if tissue-specific secretion of laminins is required for migration and midgut MET. In wild-type cells, we found two basal peaks of LanB2: one from LanB2 concentrated at the endoderm/mesoderm interface (Fig. 3 j, P1; and Fig. S5, Peak 1), and one from LanB2 localized to the basal side of the mesoderm (Fig. 3 j, P2; and Fig. S5, Peak 2). We found that expression of Sar1DN in mesoderm cells reduces the concentration of LanB2 at both the endoderm/mesoderm interface and at the basal side of the mesoderm (Fig. 3 h, quantified in Figs. 3 j and S5; P1 appears as a broad diffuse peak of FWHM 2.4 µm, and P2 is either absent or greatly reduced). When the CopII pathway is blocked in just the midgut cells alone, LanB2 levels at the endoderm/mesoderm interface are reduced (Fig. 3 i, red arrowheads, quantified in Fig. 3 j and S5; P1 is either absent or greatly reduced), while more diffuse levels of LanB2 can still be seen on the opposite side of the visceral mesoderm (Fig. 3 i, black arrowheads, quantified in Figs. 3 j and S5; P2 FWMH increases from 1 µm in wild type to 1.8 µm). This suggests that laminins from both midgut cells and the mesoderm contribute to the endodermal/mesodermal interface.

To investigate if laminin secretion from both midgut cells and mesoderm is required for midgut morphogenesis, we examined embryos expressing Sar1DN in either midgut cells or the mesoderm alone for defects in migration and repolarization. We found that in both cases, midgut migration is delayed, leading to a mispositioning of the ICP cells, as seen in both *LanB1* and CopII pathway mutants (Fig. 3, k and l, arrows), as well as gaps between the anterior and posterior midgut rudiments (Fig. 3, k and l, arrowheads). This suggests that the secretion of laminins from both midgut cells themselves and the mesoderm is required for midgut migration. However, when examining the midgut epithelium for its ability to repolarize, we found markedly different phenotypes. When laminin secretion is blocked in the midgut cells alone, we found that the midgut forms a monolayer of columnar-shaped cells, although the apical side is not as straight and ordered as in wild type (Fig. 3 m). βPS integrin localizes to the basal and apical surfaces of midgut cells as in wild type, albeit at slightly lower levels (Fig. 3, m and n3; quantified in Figs. 3 o and S2). E-Cad and Baz levels at the apical side are lower than in wild type, and they do not appear as tightly localized, but can be seen spread throughout the lateral border of the cells (Fig. 3, m and n; quantified in Figs. 3 o and S2). When laminin secretion is blocked in the mesoderm, we found more severe defects in the midgut MET: the majority of cells are rounded and multilayered; Baz, E-Cad, and βPS are found delocalized throughout the midgut cells (Fig. 3, p and q; quantified in Figs. 3 r and S2). Confocal images show a loss of βPS localized to the endoderm/mesoderm interface and diffuse βPS staining throughout the visceral mesoderm (Fig. 3, p, q3, and r; basal peak of βPS is narrow and then broadens out). These data suggest that while laminin secretion from both midgut cells and the mesoderm is required for the normal migration of endoderm cells, it is laminin secretion from the mesoderm that is predominantly required for formation of a monolayer of columnar-shaped cells and the establishment of apicobasal polarity.

### α Laminin subunits are expressed in a tissue-specific manner, and both are required for normal midgut migration and MET

**Figure 4.**
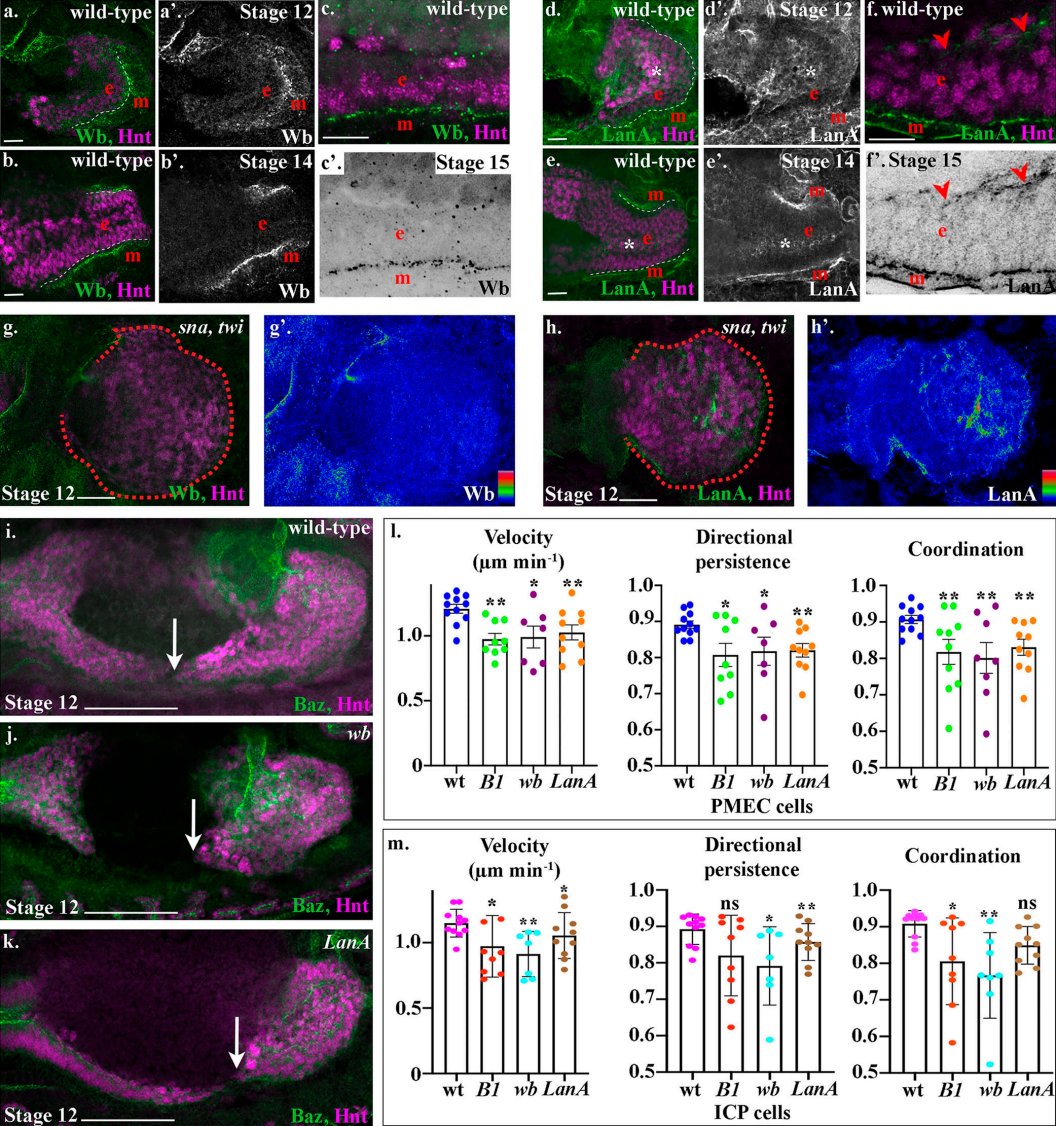
***Drosophila***** laminin heterotrimers are expressed in a tissue-specific manner, and both are required for midgut cell migration.(a–f)** Wild-type embryos mid-migration (stage 12; a and d), after migration (stage 14; b and e), or after MET (stage 15; c and f). The red e denotes the endoderm, m denotes the mesoderm, and the posterior midgut is delimited by dashed white lines. **(a–f)** Both Wb and LanA localize to the endoderm/mesoderm border throughout midgut migration and repolarization. **(d–f)** LanA is also found surrounding the ICP cells as they migrate (d and e, asterisks) and at low levels at the apical surface of the midgut cells (f, arrowheads). **(g and h)** Stage 12 *sna,twi* mutant embryos, the posterior midgut is demarked by dashed red lines. Colorimetric readouts of Wb (g′) and LanA (h′) levels on a scale of 0 to 255 (g and h). In the absence of the mesoderm, Wb staining is completely lost from the midgut (h). In contrast, LanA can still be found around midgut cells (h). **(i–k)** Stage 12 wild-type (i), *wb* (j), and *LanA* (k) embryos. Midgut cell migration is delated in both *wb* (j) and *LanA* (k) mutants. Arrows point to the tip of the posterior midgut. **(l and m)** Velocity, directional persistence, and coordination values calculated from videos of wild-type (*n* = 11), *LanB1* (*n* = 9), *wb* (*n* = 7), and *LanA* (*n* = 10) mutant embryos. *n* represents the average of a minimum of 15 cells in one embryo (shown as a dot). Data are presented as mean ± SEM. *, P ≤ 0.05; **, P ≤ 0.01 by unpaired two-tailed *t* test comparing each individual condition to wild type. One-way ANOVA tests between *LanB1*, *wb*, and *LanA* showed no significant difference in any of the conditions (PMEC velocity, P = 0.81; PMEC directional persistence, P = 0.94; PMEC coordination, P = 0.83; ICP velocity, P = 0.35, ICP directional persistence, P = 0.35, ICP coordination, P = 0.23; see Table S1 for raw data). Confocal images are oriented with the anterior to the left and posterior to the right. Scale bars, 20 µm (a, b, d, e, g, and h), 10 µm (c and f), and 50 µm (i–k).

We sought to understand why midgut cells show a different phenotype depending on whether laminin secretion is blocked cell intrinsically or from the neighboring mesoderm. As there could be tissue-specific requirements for particular laminin heterotrimers, we examined where specific α laminin subunits were expressed. Wb is very tightly localized to the endoderm/mesoderm interface during midgut migration, when the first indications of MET are apparent (Fig. 4 a), and continues to be strongly expressed and localized to the basal side of midgut cells throughout tissue morphogenesis (Fig. 4, b and c). During migration, LanA is also expressed at the endoderm/mesoderm interface but can additionally be found surrounding the ICPs, which are located on the opposite side of the PMECs from the mesoderm (Fig. 4, d and e, asterisks). After repolarization, LanA remains strongly localized at the endoderm/mesoderm interface but also localizes at low levels to the apical domain of the PMECs (Fig. 4 f, arrowheads). In mutants that completely lack mesodermal derivatives, Wb is completely lost from around the midgut cells (Fig. 4 g), whereas LanA remains expressed throughout the midgut (Fig. 4 h). These data suggest that the lamininW heterotrimer containing Wb is secreted by the mesoderm, whereas the lamininA heterotrimer is primarily secreted by the endoderm cells.

To assess the relative requirement for each of the laminin heterotrimers for midgut morphogenesis, we first examined midgut migration in *wb* and *LanA* mutants. We found that migration is severely delayed in both *wb* and *LanA* mutants, with gaps between the anterior and posterior midgut rudiments present at an embryonic stage when migration and fusion should be complete (Fig. 4, i–k). To investigate the precise requirement of Wb and LanA for the migration of midgut cells, we tracked the nuclei of subsets of PMEC and ICP cells in *wb* and *LanA* mutant embryos and compared them with *LanB1* mutants. We found that loss of either Wb or LanA leads to a slower, less directional, and less coordinated migration of both PMECs and ICPs (Fig. 4, l and m; Fig. S3; and Videos 5 and 6). Moreover, there was no significant difference in the behavior of midgut cells in either *wb* or *LanA* mutant embryos when compared with *LanB1* mutants, where there is a complete loss of laminin heterotrimers (Fig. 4, l and m; one-way ANOVA, all P values >0.5). This suggests that secretion of both Wb from the mesoderm and LanA from the endoderm is required for efficient midgut migration.

**Video 5.** **An example of a time-lapse video that was used for the tracking of migrating PMG cells in *wb* mutant embryos.** PMG cell nuclei were labeled by StingerGFP. The video begins at the initiation of germband retraction, frames are taken every 2 min, and a single z-slice is shown. Videos are tracked in 4D over multiple z-slices of 1.5-µm thickness. Playback speed, 3 frames/s.

**Video 6.** **An example of a time-lapse video that was used for the tracking of migrating PMG cells in *lanA* mutant embryos.** PMG cell nuclei were labeled by StingerGFP. The video begins at the initiation of germband retraction, frames are taken every 2 min, and a single z-slice is shown. Videos are tracked in 4D over multiple z-slices of 1.5-µm thickness. Playback speed, 3 frames/s.

### Wb acts as an upstream cue for midgut MET

**Figure 5.**
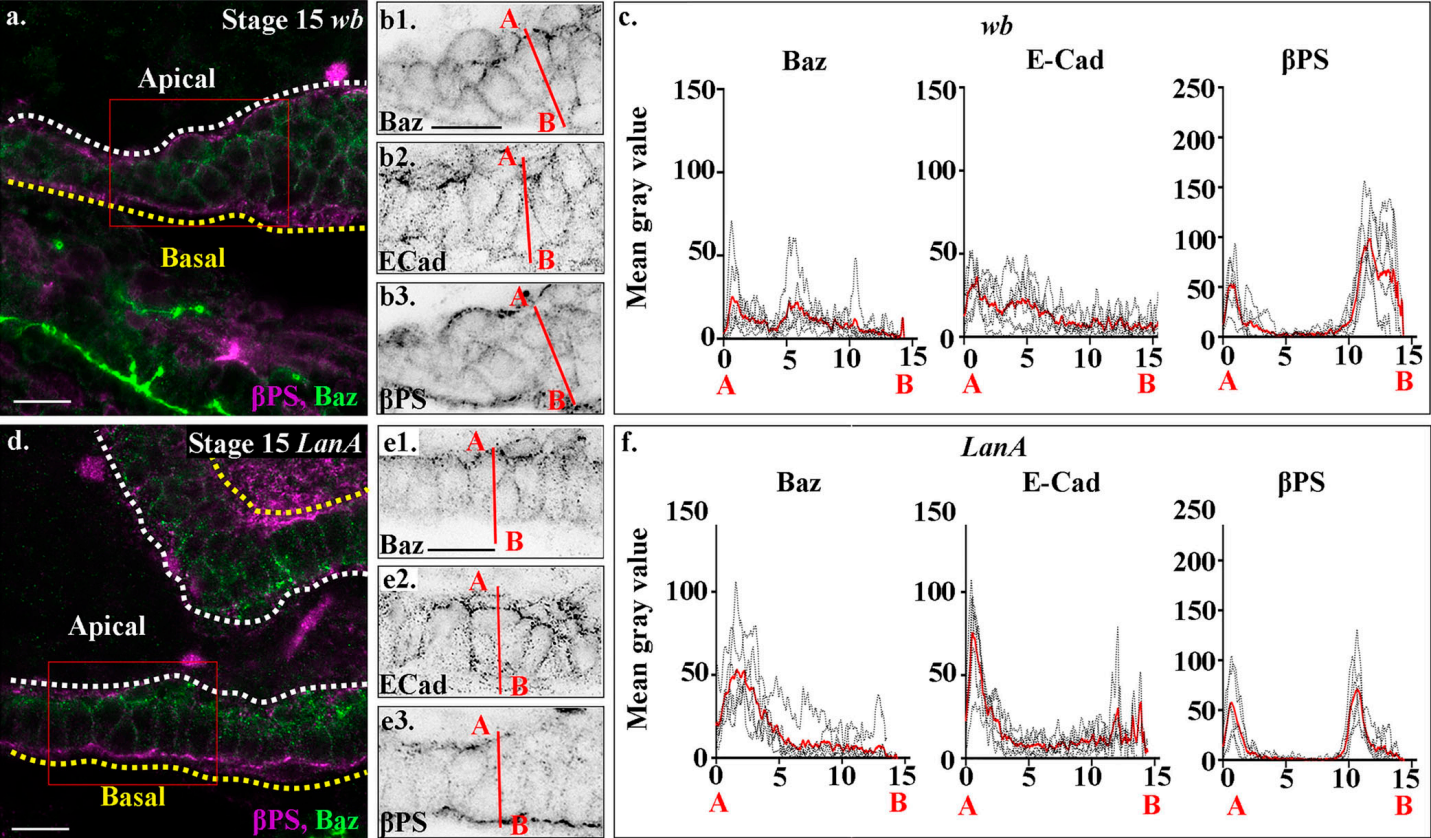
**Wb and LanA play distinct roles in midgut MET.(a–f)** Midgut cells in stage 15 *wb* (a and b) and *LanA* (d and e) mutant embryos; plots of the average fluorescence intensity (c and f, represented as mean gray value) of Baz, E-Cad, and βPS in stage 15 midgut cells mutant for *wb* (c) and *LanA* (f) measured along the apical (A) to basal (B) axis of a cell (represented in b and e by red line). Each *n* represents the average of 10 cells measured in one embryo (black dotted lines). *n* = 6 per condition, the mean for each condition is plotted in red. **(a–c)** In *wb* mutants, midgut cells fail to form an epithelial monolayer, and Baz (b1), E-Cad (b2), and βPS (b3) are found delocalized throughout the cells. **(c)** Accordingly, the apical peaks of Baz and E-cad are lost in *wb* mutants. There is just a small peak of βPS apically and a low broad peak basally, which correlates with diffuse βPS throughout the mesoderm layer. **(d–f)** In *LanA* mutants, midgut cells form a disorganized-looking monolayer of columnar or wedge-shaped cells, with βPS integrins localized to the apical and basal sides of the cells (e3), although quantification of levels reveals the peaks of βPS to be half that in wild type (f). In contrast, Baz (e1) and E-Cad (e2) are no longer restricted to the apical side of the cells (d and e) and are found in lower levels throughout the lateral membranes (d and e). Plots of the average fluorescence intensity (f) reveal the apical peak of Baz to be half that of wild-type cells and much broader, and a lower apical peak of E-Cad. White dashed lines in a and b indicate the apical side of the midgut, and yellow lines, the basal. Red boxes in a and d depict the area of the midgut epithelium shown in b and e. Confocal images are oriented with the anterior to the left and posterior to the right. Scale bars, 10 µm.

We next assessed the ability of midgut cells to undergo MET in *wb* mutants. We found a striking phenotype, with midgut cells completely failing to form a monolayer and repolarize after migration. In *wb* stage 15 embryos, posterior midgut cells are rounded and multilayered (Fig. 5 a), with Baz, E-Cad, and βPS found mislocalized throughout the cells (Fig. 5, a and b; quantified in Figs. 5 c and S2). Confocal images show a loss of βPS localized to the endoderm/mesoderm interface and diffuse βPS staining throughout the visceral mesoderm (Fig. 5, a and b). Accordingly, the FWHM of the basal βPS peak increases from the 1.3 µm seen in wild-type cells to 2 µm (Figs. 5 c and S2). This contrasts with the phenotype seen in *LanA* mutants, where midgut cells form a monolayer of columnar-shaped cells, although the apical side is not as straight and ordered as in wild type (Fig. 5 d). βPS integrin localizes to the basal and apical surfaces of midgut cells as in wild type, but at far lower levels than in wild type (Fig. 5, d and e3; quantified in Figs. 5 f and S2). E-Cad and Baz levels at the apical side are also almost half those in wild type, and they do not appear as tightly localized but can be seen spread throughout the lateral border of the cells (Fig. 5, d and e; quantified in Figs. 5 f and S2). Quantifications show that the phenotype of *wb* mutants is remarkably similar to when Sar1DN is expressed in the mesoderm (compare Fig. 5 c with Fig. 3 r; Fig. S2), as is the phenotype of *LanA* mutants to when Sar1DN is expressed in the endoderm (compare Fig. 5 f with Fig. 3 o; Fig. S2). Taken together, these findings suggest that *wb* secretion from the mesoderm is absolutely required for midgut cells to form a polarized monolayer of columnar-shaped cells, while LanA secretion from the endoderm cells themselves is required for their robust repolarization.

**Figure 6.**
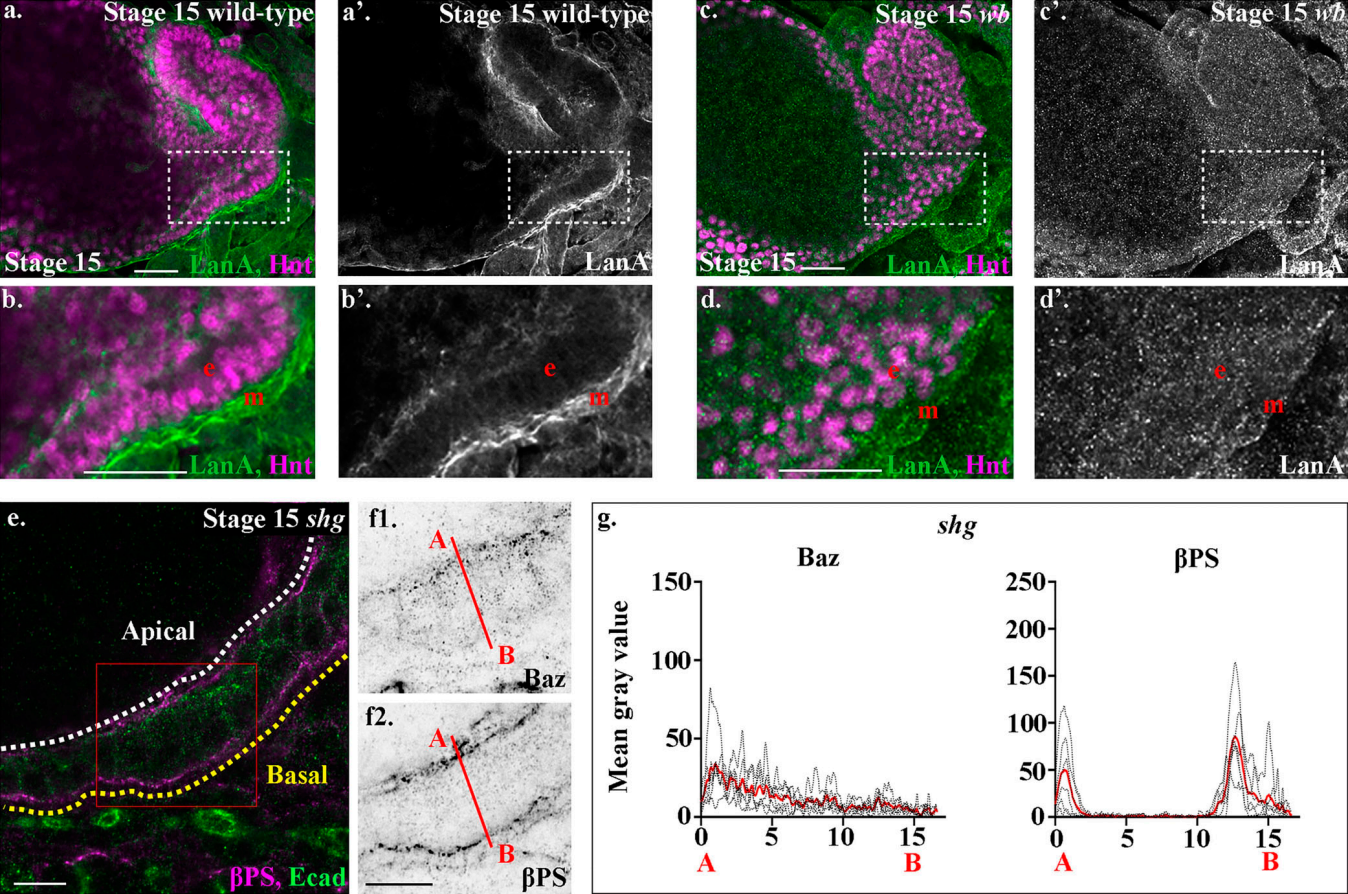
**Wb acts as an upstream cue for midgut polarity.(a–d)** Stage 15 wild-type (a and b) and *wb* (c and d) embryos. Dashed boxes in a and c show the areas enlarged in b and d. In *wb* mutants, the strong LanA localization to the endoderm/mesoderm border seen in wild type (a and b) is lost (c and d). **(d)** Many LanA punctae are found inside midgut cells in *wb* mutants. **(e–g)** In *shg* mutants, midgut cells form a disorganized-looking monolayer of columnar or wedge-shaped cells, with βPS integrins localized to the apical and basal sides of the cells (f2). In contrast, Baz (f1) is not localized to the apical side of the cells, but shows low levels of diffuse staining throughout. **(g)** Plots of the average fluorescence intensity (represented as mean gray value) of Baz and βPS in stage 15 midgut cells mutant for *shg* measured along the apical (A) to basal (B) axis of a cell (represented in f by red line). Each *n* represents the average of 10 cells measured in one embryo (black dotted lines). *n* = 6 per condition; the mean for each condition is plotted in red. The apical peaks of Baz are lost in *wb* mutants, and the levels of both apical and basal peaks of βPS are reduced to just under half of wild type. White dashed line in e indicates the apical side of the midgut, and the yellow line, the basal. Red box in e depicts the area of the midgut epithelium shown in f. Confocal images are oriented with the anterior to the left and posterior to the right. Scale bars, 20 µm (a–d) and 10 µm (e and f).

To dissect whether Wb acts upstream of, or in parallel to, LanA in mediating midgut MET, we next examined LanA localization in *wb* mutants. We found that in *wb* mutants, LanA is completely absent from the endoderm/mesoderm interface, and that midgut cells are filled with LanA-positive puncta (Fig. 6, a–d). Thus, in *wb* mutants, midgut cells are not able to secrete LanA. These data suggest that secretion of Wb by the mesoderm acts as an upstream cue for midgut MET and is required for the polarized secretion of LanA. This secretion of LanA by midgut cells is in turn required to reinforce their polarity and to restrict the localization of apical proteins.

E-Cad–mediated cell–cell adhesion has previously been shown to be required for formation of the embryonic midgut epithelium (Tepass and Hartenstein, 1994). If Wb acts as an upstream cue for midgut repolarization and the formation of a columnar monolayer of cells, we reasoned that βPS integrin would localize correctly in mutants for *shotgun (shg)*, the gene encoding *Drosophila* E-Cad. It is not possible to analyze midgut morphogenesis in the complete absence of E-Cad, due to the requirement for its maternal contribution during oogenesis and very early embryonic development. We therefore focused our analysis on the strong allele *shg^G317^*, which shows a phenotype stronger than the zygotic null mutant alleles, likely due to a dominant-negative affect on the maternal contribution (Tepass et al., 1996). We previously used this allele to uncover a role for E-Cad in mediating cohesive migration of the midgut cells (Campbell and Casanova, 2015). In *shg^G317^* mutant embryos, we found that despite completely failing to localize Baz correctly to the apical domain (Fig. 6, e–g; and Fig. S2), midgut cells in *shg* mutant embryos localize βPS both apically and basally, albeit at lower levels that are reminiscent of those seen in *LanA* mutants (Fig. 5, a–d). This suggests that basal cues are localized independently of E-Cad–mediated cell–cell adhesion, and fits with a model where the basal surface of the midgut is defined by interactions between integrins and Wb that is secreted by the adjacent mesoderm.

### Wb is required for the distinct localization of αPS integrins, whereas LanA is required to reinforce their levels

**Figure 7.**
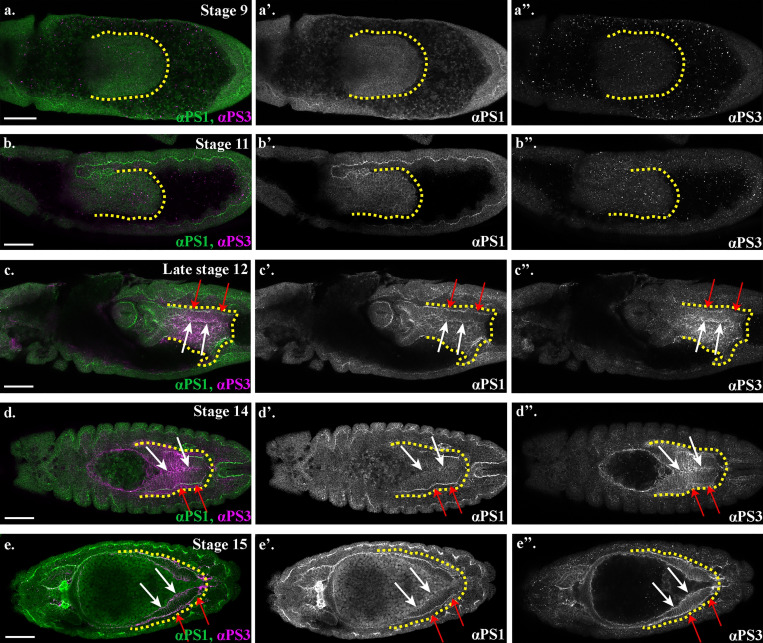
**αPS1 and αPS3 integrin subunits become expressed in the midgut during MET and localize to distinct domains.(a–e)** Wild-type embryos stained for αPS1 (green) and αPS3 (magenta). White arrows indicate αPS3 in the apical domain of the midgut cells, and red arrows point out αPS3 in the basal region. Yellow dotted lines outline the posterior midgut. Scale bars, 50 µm.

**Figure 8.**
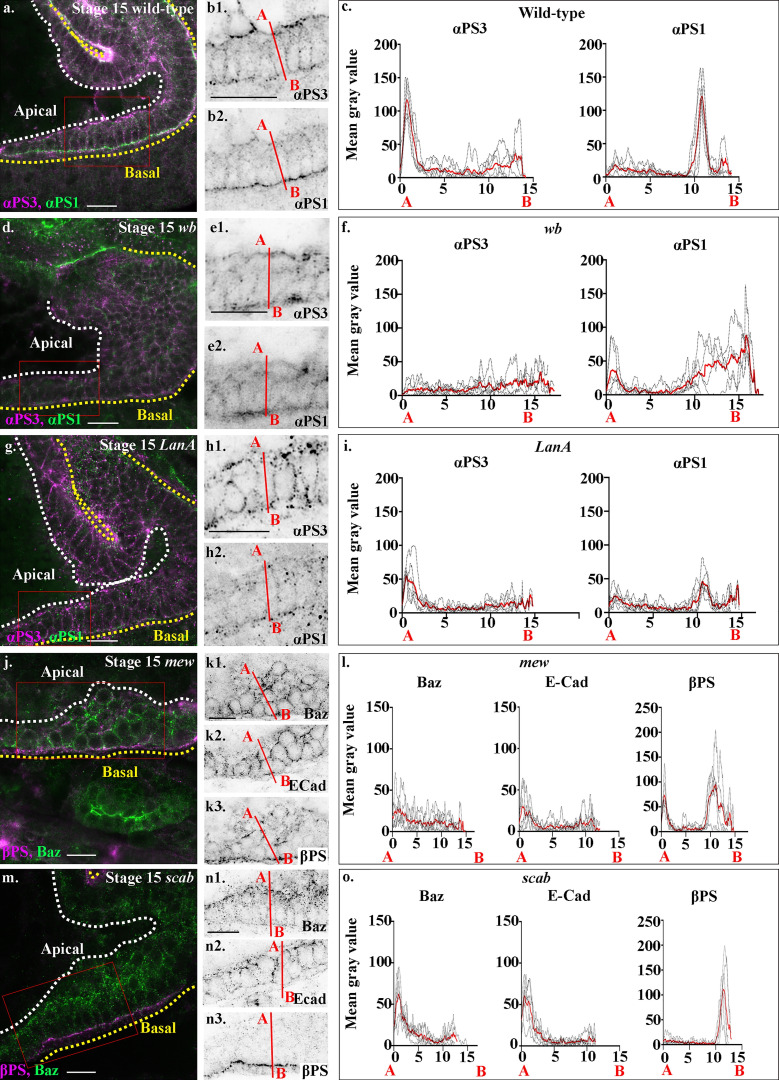
**Loss of the α integrin subunits αPS1 or αPS3 gives distinct MET phenotypes.** Stage 15 wild-type (a and b), *wb* (d and e), *LanA* (g and h), *mew* (mutant for αPS1, j and k), and *scab* (mutant for αPS1, m and n) stained for αPS1 and αPS3 (a, b, d, e, g, and h); or Baz, βPS (j, k, m, and n) and E-Cad (k2 and n2). **(c, f, i, l, and o)** Plots of the average fluorescence intensity (represented as mean gray value) of αPS1 and αPS3 (c, f, and i) or Baz, E-Cad, and βPS (l and o) in either wild-type midgut cells (c) or midgut cells mutant for *wb* (f), *LanA* (i), *mew* (l), or *scab* (o). Plots are measured along the apical (A) to basal (B) axis of a cell (represented in b, e, h, k, and n by red line). Each *n* represents the average of 10 cells measured in one embryo (black dotted lines). *n* = 6 per condition; the mean for each condition is plotted in red. **(a–c)** αPS1 and αPS3 show distinct localizations in midgut cells, with αPS1 (b2 and c) localizing basally and αPS3 (b1 and c) apically (the small basal peak of αPS3 is in the mesoderm). **(d–f)** In *wb* mutants, the polarized localization of both αPS1 (e2 and f) and αPS3 (e1 and f) is lost. **(g–i)** In *LanA* mutants, αPS3 levels (h1) are lowered at the apical domain (i), and it shows a more diffuse staining in the cell. While αPS1 localizes predominantly to the basal domain (h2), its levels are just over half that in wild type (i). **(j–l)** In mutants for αPS1, *mew,* the midgut looks very similar to in *wb* mutants: cells fail to form an epithelial monolayer, and Baz (k1), E-Cad (k2), and βPS (k3) are found delocalized throughout the cells. **(l)** Accordingly, the apical peaks of Baz and E-Cad are lost in *mew* mutants. There is just a small peak of βPS apically and a low broad peak basally which correlates with diffuse βPS throughout the mesoderm layer. **(m–o)** In *scab* mutants, the midgut looks very similar to that in *LanA* mutants: midgut cells form a disorganized-looking monolayer of columnar or wedge-shaped cells. **(n and o)** Baz and E-Cad levels reduced, with their apical peaks half the height of those in wild type. βPS integrin staining is completely lost from the apical side of the cell, but only slightly reduced on the basal side (n3 and o). White dashed lines in a, d, g, j, and m indicate the apical side of the midgut, and yellow lines, the basal. Red boxes in a, d, g, j, and m depict the area of the midgut epithelium shown in b, e, h, k, and n. Confocal images are oriented with the anterior to the left and posterior to the right. Scale bars, 10 µm.

**Figure 9.**
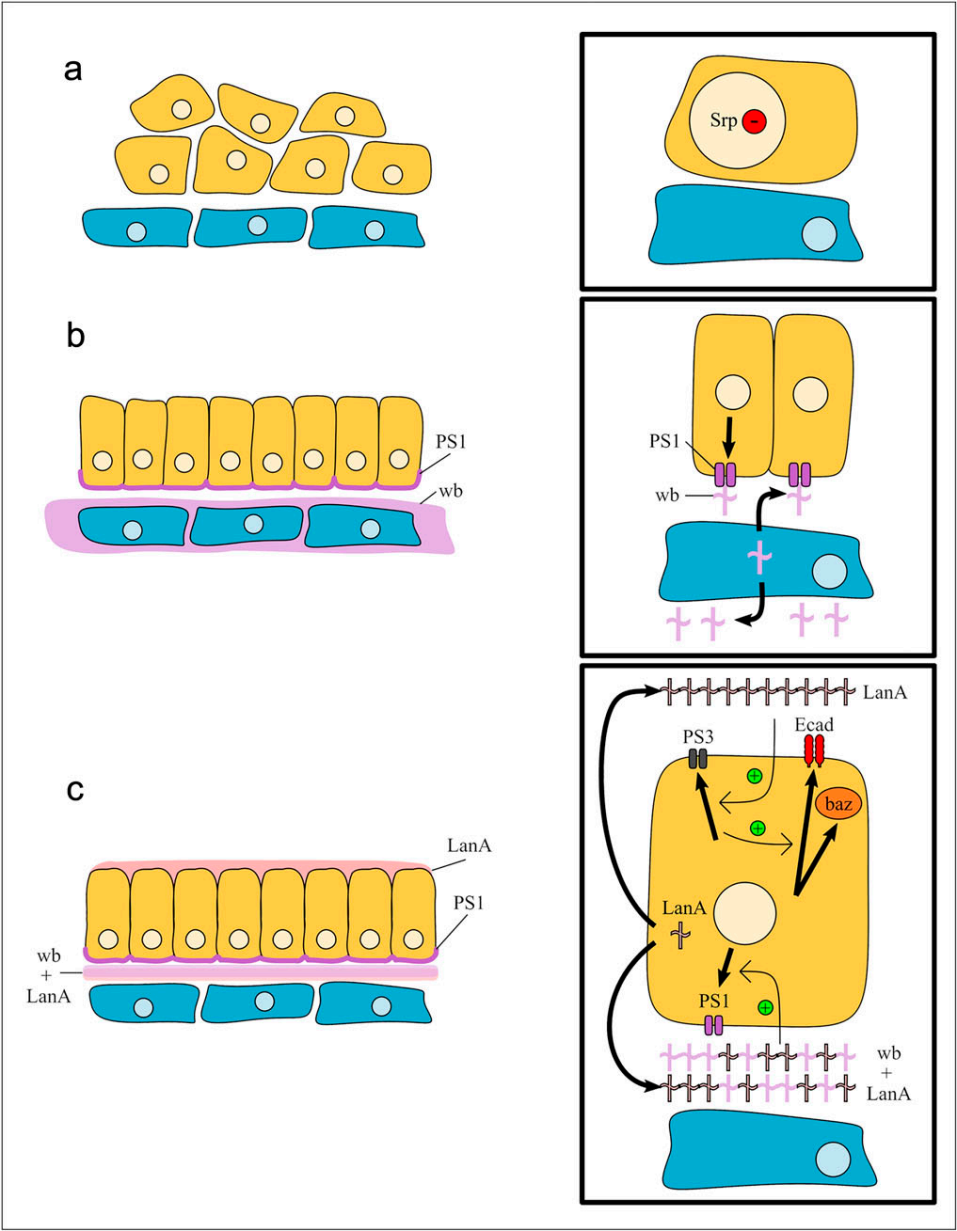
**Model of *Drosophila* midgut MET. (a)** Serpent is down-regulated in endoderm cells independently of contact with the mesoderm. **(b)** The mesoderm secretes Wb. αPS1 localizes to the side of the endoderm that contacts the mesoderm. This defines the basal side of the cells and is required for the cells to establish apicobasal polarity and form a monolayer of columnar-shaped cells. **(c)** αPS3 localizes to the apical side of midgut cells, and this is required for the robust localization of E-Cad and Baz to the apical side of the cell. Finally, secretion of LanA by the midgut cells acts to reinforce αPS1 basally and αPS3 apically, and thereby ensure robust polarity.

If Wb and LanA both bind to integrin receptors in the midgut cells, then this raises the question of how they differentially contribute to MET and the repolarization of the cells. The specific function of PS integrins during embryogenesis has previously been demonstrated to reside in the α subunit extracellular domain (Martin-Bermudo et al., 1997). We therefore decided to investigate the specific localization and function of the two α PS integrin subunits, which have been previously been described to be expressed in the midgut cells (Brower et al., 1995; Stark et al., 1997) and to play partially redundant roles in mediating midgut migration (Martin-Bermudo et al., 1999). αPS1 and αPS3 subunits first become expressed in the midgut mid-migration, correlating with the earliest stages of MET (Fig. 7, compare with Fig. S1). Strikingly, staining for αPS1 and αPS3 reveals that αPS subunits localize to distinct regions of midgut cells, with αPS3 localizing tightly to the apical domain and αPS1 to the basal side of the cell (Fig. 8, a and b; quantified in Fig. 8 c), suggesting that it is αPS1, rather than αPS3, that binds to Wb. Accordingly, mutants for *mew*, the gene encoding αPS1, phenocopy *wb* mutants (compare Fig. 8, j–l with Fig. 5, a–c; Fig. S2), and in *wb* mutants, the polarized localization of both αPS1 and αPS3 are lost (Fig. 8, d–f; and Fig. S5, FWHM for αPS1 peak increases from the 0.8 µm in wild type to 2 µm). Conversely, in *LanA* mutants, αPS3 and αPS1 both show some degree of polarization within the midgut cells (Fig. 8, g–i; and Fig. S5, FWHM for αPS1 nor αPS3 peaks are not significantly different from those in wild type). However, the levels of localized αPS3 and αPS1 are low, and quantification reveals the peaks to be less than half those seen in wild type (compare Fig. 8 i with Fig. 8 c; Fig. S5). These results suggest that the secretion of LanA is required to reinforce αPS1 levels at the basal domain of midgut cells, and αPS3 at the apical. Apical αPS3, in turn, is required for the correct levels of E-Cad, Baz, and βPS at the apical domain, as in mutants for *scab* (which lack αPS3), E-Cad, and Baz levels at the apical side are almost half those in wild type, and the apical peak of βPS is completely lost (Fig. 8, m–o; and Fig. S2). Taken together, these results suggest a model where Wb secretion by the mesoderm leads to αPS1 recruitment to the basal domain of midgut cells (Fig. 9). This provides an upstream cue that establishes apicobasal polarity and the formation of a monolayer of columnar-shaped cells. αPS3 is localized to the apical domain, and this is required for the robust localization of E-Cad and Baz to the apical side of the cell. Finally, secretion of LanA by the midgut cells acts to reinforce αPS1 basally and αPS3 apically, and thereby ensure robust polarity (Fig. 9).

## Discussion

Despite its key roles during development and cancer progression, very little is known about the molecular mechanisms orchestrating MET, and in particular how these mechanisms relate to those driving the reverse process, EMT. Here we demonstrate, in vivo, that while MET requires the down-regulation of EMT-TFs, this in itself is not sufficient for MET to take place. Additional extracellular cues are also required, and in the case of the *Drosophila* midgut, are provided by LanW secretion by the adjacent mesoderm, a specific laminin heterotrimer containing the laminin α1,2 chain, Wb. Wb acts as an upstream cue for midgut MET and is required for the secretion of the laminin heterotrimer containing laminin α3,5 chain, LanA, from the midgut cells themselves. LanA in turn is required for the robust repolarization of the midgut cells. While laminins have previously been shown to play nonautonomous roles in orienting polarity in vitro (O’Brien et al., 2001) and in vivo during pharynx development in *Caenorhabditis elegans* (Rasmussen et al., 2012), this work reveals additional unexpected cell-autonomous roles for laminins in midgut migration and repolarization, as well in the specificity of the laminin isoforms required. Here we first demonstrate that, rather than simply migrating on a trail secreted by neighboring cells, midgut cells appear to contribute their own tracks for migration. Second, we demonstrate a distinct role for the two laminin heterotrimers in the reepithelization and repolarization of the embryonic midgut epithelium.

This dual requirement for the down-regulation of an EMT-TF and exposure to tissue-specific extracellular cues may represent a conserved mechanism for MET. Vertebrate somites are precursors of the vertebrae in the spine, and defects in somitogenesis are linked to congenital scoliosis (Eckalbar et al., 2012; Pourquié, 2011). Studies of normal somitogenesis are providing important insights into the underlying causes of congenital disorders. Somite formation relies on paraxial mesoderm undergoing MET, after cells have migrated away from the primitive streak. As they migrate, the cells maintain high levels of Snail1 and Snail2, which are then down-regulated as they move to regions with lower levels of FGF, coinciding with acquisition of epithelial characteristics. Similar to overexpression of Serpent in *Drosophila* midgut cells, overexpression of Snail in paraxial mesoderm blocks MET (Dale et al., 2006). While it has not been directly investigated, there is evidence to suggest that this might not be sufficient for MET to occur. Removal of the tissue that lies adjacent to the somites, the surface ectoderm, results in a failure of paraxial mesoderm cells to undergo MET (Correia and Conlon, 2000; Šošić et al., 1997). Loss of MET is unlikely to be due to a failure in *Snail* gene down-regulation, as Snail regulation occurs downstream of changes in FGF levels (Dale et al., 2006) and should therefore be independent of ectodermal interactions. In an intriguing parallel to the role of the mesoderm during *Drosophila* midgut MET, the chick ectoderm appears to be required for the secretion of the ECM component fibronectin, which is required for the reepithelization of the somites via integrin-mediated adhesion (Girós et al., 2011; Rifes et al., 2007).

The growth of an overt secondary metastasis represents the final and most deadly phase in the malignant progression of a tumor, and increasing evidence suggests that METs play a central role in this process. This makes it important to understand how MET relates to EMT. Indeed, pharmaceutical companies have been seeking ways to block EMT and thus prevent delamination of the primary tumor. If EMT and MET are related, then inhibiting EMT actually risks favoring MET and ectopic colonization by cells that have already metastasized away from the primary tumor. Our results suggest that down-regulating the activity of an EMT-TF and MET induction may be separable events. This implies that rather than directly promoting MET, blocking EMT may instead prime circulating cells to undergo MET when they meet an inductive cue in the surrounding environment. Accordingly, there is emerging evidence pointing to a role for extracellular cues from the metastatic niche that induce MET in the colonizing cells, such as Versican in the metastatic lung niche (Gao et al., 2012), and activated fibroblasts in the lungs (Del Pozo Martin et al., 2015). While down-regulation of EMT-TFs has been observed in both cases, other pathways such as the polarized localization/activation of integrins may also be required. Combined targeting of these pathways with therapies that prevent EMT may avert the potentially detrimental effects of blocking EMT alone.

Using a range of approaches including live imaging and high-resolution confocal imaging, we have dissected the intrinsic and external cues that govern MET during midgut migration, identifying a role for specific laminin heteromers in guiding this important process in early development. Future studies will be required to dissect the molecular mechanisms acting downstream of these interactions, which will likely lead to exciting new diagnostic and therapeutic targets for metastatic cancer.

## Materials and methods

### Fly strains and genetics

Standard procedures were used for *Drosophila* maintenance and experiments. Flies were grown on standard fly food supplemented with live yeast at 25°C. Details for all genotypes and transgenes can be found in Flybase (http://flybase.org) or in references listed here. Unless otherwise noted, stocks were obtained from the Bloomington Drosophila Stock Center [BDSC]). Wild-type embryos were from *yw* stocks. The Gal4/UAS system (Brand and Perrimon, 1993) was used to drive the expression of transgenes. Transgenes were driven in the posterior midgut using either the hkb-Gal4 (gift from Helen Skaer [University of Cambridge, Cambridge, UK], drives transgenes in the entire *hkb* expression domain) or the 48Y-Gal4 (BDSC 4935) drivers alone, or recombined with UAS-StingerGFP (BDSC 84277); they were driven in the muscle using a Twist-Gal4 (BDSC 914). Flies with the following genotypes were used: *sna^18^*,*twi^3^* (BDSC 3299), *sar1^05712^* (BDSC 11669), *sec23^9G^* (BDSC 3094), *sec16^A^* (BDSC 52390), UAS-Sar1DN (gift from Christos Samakovlis, Stockholm University Stockholm, Sweden; Tsarouhas et al., 2007), UAS-*sar1*^T38N^ (a dominant-negative form of *sar1*), *LanB1^IP3^* (gift from Maria Dolores Martín-Bermudo [Centro Andaluz de Biología del Desarrollo, Seville, Spain], an amorphic allele, described in Urbano et al. [2009]), *wb*^09437^ (BDSC 12362), *LanA^9-32^* (gift from Talila Volk [Weizmann Institute of Science, Rehovot, Israel], an amorphic allele, described in Yarnitzky and Volk [1995]), *shg^317^* (gift from Ulrich Tepass [University of Toronto, Toronto, Canada], *shg^317^* shows a phenotype stronger than the zygotic null mutant alleles, likely due to a dominant-negative affect on the maternal contribution described in Tepass et al. [1996]), *serpentHemo::3xmCherry* (BDSC 78358), *mew^M6^* (BDSC 1483), and* scab^l7^*(BDSC 6483).

### Immunohistochemistry, fixed image acquisition, and analysis

Embryos were fixed, mounted, and staged using standard techniques. Embryos were staged according to the Hartenstein atlas. Antibodies used were as follows: mouse anti-αPS1 (DK.1A4; 1:50); αPS3 (1:1,000; gift from Shigeo Hayashi, Riken Center for Developmental Biology, Kobe, Japan); rabbit anti-Baz (1:1,000; gift from Andreas Wodarz, University of Cologne, Köln, Germany); mouse anti-βPS integrin (COMPARE6G11; 1:20; Hybridoma Bank); rat anti-E-Cad (DCAD2; 1:100; Hybridoma Bank); mouse anti-FasII (ID4; 1:20; Hybridoma Bank); mouse anti-FasIII (7G10; 1:20; Hybridoma Bank); goat anti-GFP (AB6673; 1:500; Abcam); mouse anti-Hnt (1G9; 1:20; Hybridoma Bank); rabbit anti-Laminin A (gift from Stefan Baumgartner, Lund University, Lund, Sweden); rabbit anti-Laminin B2 (1:500, ab47651, Abcam); rat anti-Serpent (1:500; Campbell et al., 2018); and rabbit anti-Wb (gift of Stefan Baumgartner). For staining with αPS1, embryos were fixed in 4% PFA for 30 min, mounted on double-sided sticky tape in 1× PBT-0.3% BSA, and mechanically devitellinized using a 25G needle. For labeling with anti-E-Cad, embryos were fixed in 4% PFA for just 10 min. For all other stainings, embryos were fixed using standard techniques. The following Alexa Fluor secondary antibodies, all made in donkey, were used at a dilution of 1:200–1:500: anti-goat 488 (ab150129; Abcam); anti-rat 555 (ab150154; Abcam); anti-rabbit 555 (A31572; Invitrogen); anti-mouse 555 (A32773; Invitrogen); anti-rat 555 (A48270; Invitrogen); anti-mouse 647 (A31571; Invitrogen); anti-rabbit 647 (A-31573; Invitrogen); and anti-rat 647 (ab150155; Abcam). Embryos were mounted in Fluoromount-G. Confocal images were acquired using a Zeiss LSM880 with either the internal GaAsP detectors or an Airyscan detector; a Plan-Apochromat 25×/0.8 multi-immersion lens with oil or a Plan-Apochromat 63×/1.40 oil-immersion lens for fixed images; and a Plan-Apochromat 40×/1.3 oil-immersion lens for live imaging. Images taken using the Airyscan detector were processed using Zen software. Images were analyzed using ImageJ Fiji distribution software (Schindelin et al., 2012). Figures were assembled using Adobe Photoshop. All images presented in the figures are single slices taken from z-stacks through the embryo. All images are oriented with the anterior end of the embryo to the left and posterior to the right.

### Reproducibility of experiments

All experiments were repeated multiple times as listed below. For Fig. 1, c–e and h–j; Fig. 2, h–j; Fig. 3, h–j and m–r; Fig. 5; Fig. 6, e–g; and Fig. 7, a minimum of 10 cells were measured per embryo, and 6 embryos were measured for each protein, per condition; representative images are shown. For Fig. 1; Fig. 2, f and g; Fig. 3, f and g; and Fig. 4, l and m; an average of 15 of each cell type per video were tracked, corresponding to some 300 nuclei positions over time per embryo. A minimum of six videos were analyzed per condition. For all other figures, the numbers of experiments are listed below; the first number indicates the number of times the experiment was repeated, and the second number indicates the number of embryos analyzed; representative images are shown in the figures: Fig. 1, a–d (3, >40); Fig. 2, a, d, and e (3, >40); Fig. 3, a–c (3, >40, all *sar1* embryos showed gaps and/or mispositioned ICPs, the migration phenotype is quantified in Fig. 3, f and g), Fig. 3 d (4, >40), Fig. 3 e (4, >40, large intracellular puncta were seen in all embryos analyzed), Fig. 3, k and l (3, >40, all embryos showed gaps and mispositioned ICPs); Fig. 4, a–h (3, >40), Fig. 4, i–k (3, >40, migration phenotype is quantified in Fig. 4, l and m); and Fig. 6, a–d (3, >40, all *wb* embryos show a loss of LanA at the endoderm/mesoderm interface and large amounts of intercellular puncta of LanA in midgut cells).

### Quantification of protein localization and levels

Localization of Srp, Baz, E-Cad, βPS, αPS1, αPS3, and LanB2 was measured using ImageJ Fiji distribution software (Schindelin et al., 2012). Two-color images were split into individual channels, converted to 8-bit/grayscale images, inverted, background subtracted, and thresholded. Background subtraction was performed using the sliding paraboloid method. Thresholding was done using the stacked histogram method set at a constant value (3%) for each image, enabling comparisons of mean gray values, representing relative protein levels, between images. For Srp, the oval tool was used to outline each midgut nuclei, and the mean gray value was measured. 30 nuclei per embryo were analyzed and averaged to generate an overall mean gray value per embryo; 6 embryos were analyzed per condition. For all other proteins, the straight line tool was used to draw a line bisecting individual cells in an apical to basal direction, ensuring the width of the line filled the area of a cell without touching/intersecting the lateral membranes. Lines were drawn in the plane of the cell from 1 µm above the apical membrane of midgut cells to 1 µm below the basal surface of the visceral mesoderm in a single z-slice (Fig. S2). The length of each cell within the ventral posterior region of the midgut was measured (boxed region in Fig. S2). A minimum of 10 cells with similar lengths (±1 µm) were measured per embryo. Lines were drawn on raw images to ensure the lines were drawn precisely and accurately and saved as regions of interest. After the background was subtracted, images were inverted and thresholded. The stack histogram threshold method enables simultaneous separation of background and signal pixels as well as normalization across images. Then, regions of interest were applied to the thresholded images and measured using the plot profile function in ImageJ.

The plot profile function was used to determine the mean gray value per horizontal line along the length of a single plotted line, using a standard line width of 60 pixels, providing local averaging. This enabled the majority of the width of a single cell to be measured, while excluding the lateral membranes. A minimum of 10 cells per embryo were analyzed and averaged to generate an overall mean gray value per embryo. Quantification of protein localization and relative expression levels was performed on six embryos per condition. Graphical representations of mean gray values were generated using GraphPad Prism 9.0.1. The average of the cells in an individual embryo is represented as a dashed black line and the overall average of all embryos per condition as a solid red line.

### Calculating peak width and peak height

FWHM was measured by calculating the width of the peak at half-maximum peak height per embryo. Peak height was measured by finding the maximum value of peaks within specific ranges of the cell length.

### Live imaging

Embryos were dechorionated using bleach, and stage 10 embryos were manually picked from an agar plate using a Zeiss fluorescent dissecting microscope. The selected embryos were dorsally or laterally oriented and mounted on a coverslip coated with heptane glue to prevent drift during imaging. A drop of Voltalef 10S oil was placed on the embryos to maintain their survival. Embryos were imaged using an inverted Zeiss LSM880, in an incubation system heated to 25°C, with a Chameleon Discovery dual output multiphoton laser, with the spectral laser tuned to 890-nm wavelength and a Plan-Apochromat 40×/1.3 oil-immersion lens. Multiposition time-lapse stacks of 20–25 µm and a z-depth of 1.5 µm were acquired at 2-min intervals over a period of 60 min. A minimum of six videos per condition were selected for analysis, and the starting point was defined as the initiation of germband retraction, which is unaffected in the different conditions.

### Time-lapse preprocessing

The cells under study can exhibit a fast-directed movement and are densely packed, making their tracking challenging. However, overall movement can be broadly estimated and compensated for, thus lowering the burden on the tracker. These procedures were performed using a custom ImageJ macro that incrementally and uniformly shifts all the images from each frame to partially cancel out the estimated overall displacement. The shifts applied to the images were stored and accounted for at a later stage.

### Nucleus tracking

Tracking of GFP-positive nuclei was performed using nuclear fluorescence signal with the Fiji plugin Trackmate. This plugin implements a blob detector based on an adjusted 3D Laplacian of Gaussian filter followed by 3D local minima detection. The candidate nuclei are selected based on intensity at the local minima and the distance to the closest local minima; if this distance is closer to the characteristic radius of the nuclei, only the strongest minima are kept. This last criterion vastly improved the results of the tracking and was implemented as a custom extension to Trackmate. To link the nuclei and build their tracks, the plugin then relies on a frame-to-frame constrained linear assignment.

### Visualization and correction of tracks

The nuclei tracks were exported from Trackmate to spreadsheets, and the original overall movement was numerically restored by accordingly shifting the positions of the detected blobs. Each track was then manually checked. To this end, we developed a custom ImageJ macro allowing the overlay of tracks originating from a specific region of interest to the original video. With this tool, we were able to follow a nucleus along a track in 3D, as the z-slice is automatically adjusted to the detected position. Tracks from ICPs and PMECs were selected according to the nuclear diameter: PMECs <3.5 µm and ICPs >5.5 µm. Only the valid tracks starting inside the specified regions and spanning the whole video were kept, yielding an average of 15 tracks of each cell type per video, corresponding to some 300 nuclei positions defined over time per embryo. For more details on each of the custom macros used in this work, and on how they can be downloaded, please see Tosi and Campbell (2019).

### Tracks statistics

From the selected tracks, the instantaneous speeds of cells were estimated from the nuclei frame-to-frame displacements. A metric of cohesion of the local movements was estimated as the correlation of a nucleus instantaneous speed direction (and magnitude) and that of the nucleus from the closest track (track distance evaluated at the first time point; Campbell and Casanova, 2015). From this information, the track average velocity, coordination, and directional persistence were derived. Finally, the average and SD of these measurements were computed over all the tracks of these measurements of all the videos from the same condition.

### Online supplemental material

Fig. S1 shows characterizations wild-type midgut MET and Srp down-regulation in wild-type and *sna,twi* embryos. Figs. S2 and S5 show FWHM and amplitude (maximum height) of fluorescence intensity peaks. Fig. S3 shows representative tracks of paths taken by PMECs and ICPs in each genotype. Fig. S4 shows that midgut migration and repolarization is perturbed in zygotic mutants for the CopII trafficking pathway. Video 1 shows a time lapse of hemocyte and midgut migration. Videos 2, 3, 4, 5, and 6 show time lapses used for tracking migrating PMG cells in wild-type, *LanB1* mutant, *sar1* mutant, *wb* mutant, and *lanA* mutant embryos, respectively. Table S1 shows raw data from cell tracking experiments.

## Supplementary Material

Table S1 shows raw data from cell tracking experiments.
