# Supplementary material for: Mesenchymal-to-epithelial transitions require tissue-specific interactions with distinct laminins
Source: J Cell Biol. 2021 May 28;220(8):e202010154. doi: 10.1083/jcb.202010154 (PMC8167899; doi:10.1083/jcb.202010154)
Supplement: Table S1 — shows raw data from cell tracking experiments. [file JCB_202010154_TableS1.docx]

**Supplementary Table 1.**

| wild-type | ICP velocity μm/min | PMEC velocity μm/min | ICP/PMEC  ratio | ICP dir persistency | PMEC dir persistency | ICP coordination | PMEC coordination |
| --- | --- | --- | --- | --- | --- | --- | --- |
| mov1 | 1.311 | 1.346 | 0.974 | 0.903 | 0.921 | 0.930 | 0.944 |
| mov2 | 1.150 | 0.962 | 1.195 | 0.912 | 0.907 | 0.923 | 0.905 |
| mov3 | 1.309 | 1.177 | 1.112 | 0.907 | 0.879 | 0.932 | 0.931 |
| mov4 | 1.170 | 1.311 | 0.892 | 0.927 | 0.876 | 0.934 | 0.881 |
| mov5 | 1.088 | 1.307 | 0.832 | 0.840 | 0.846 | 0.837 | 0.847 |
| mov6 | 1.192 | 1.243 | 0.959 | 0.925 | 0.939 | 0.942 | 0.942 |
| mov7 | 0.949 | 1.141 | 0.832 | 0.897 | 0.893 | 0.923 | 0.908 |
| mov8 | 1.094 | 1.080 | 1.013 | 0.920 | 0.870 | 0.921 | 0.881 |
| mov9 | 1.207 | 1.281 | 0.942 | 0.931 | 0.946 | 0.922 | 0.967 |
| mov10 | 1.063 | 1.246 | 0.853 | 0.846 | 0.881 | 0.853 | 0.911 |
| mov11 | 1.102 | 1.197 | 0.921 | 0.807 | 0.847 | 0.872 | 0.861 |
| average | 1.149 | 1.208 | 0.957 | 0.892 | 0.891 | 0.908 | 0.907 |
|  |  |  |  |  |  |  |  |
| *sar1* |  |  |  |  |  |  |  |
| mov1 | 0.904 | 0.772 | 1.171 | 0.710 | 0.722 | 0.724 | 0.703 |
| mov2 | 0.867 | 0.812 | 1.068 | 0.683 | 0.618 | 0.693 | 0.589 |
| mov3 | 0.921 | 1.008 | 0.913 | 0.829 | 0.851 | 0.851 | 0.864 |
| mov4 | 0.979 | 0.958 | 1.022 | 0.870 | 0.882 | 0.918 | 0.909 |
| mov5 | 0.665 | 0.701 | 0.948 | 0.800 | 0.782 | 0.791 | 0.755 |
| mov6 | 0.942 | 1.008 | 0.935 | 0.777 | 0.725 | 0.781 | 0.721 |
| average | 0.880 | 0.877 | 1.010 | 0.778 | 0.763 | 0.793 | 0.757 |
| p-value | **0.000** | **0.000** | 0.359 | **0.001** | **0.001** | **0.001** | **0.001** |
|  |  |  |  |  |  |  |  |
| *LamB1* |  |  |  |  |  |  |  |
| mov1 | 0.931 | 0.958 | 0.972 | 0.753 | 0.679 | 0.754 | 0.726 |
| mov2 | 0.874 | 0.897 | 0.974 | 0.870 | 0.752 | 0.815 | 0.717 |
| mov3 | 0.720 | 0.896 | 0.804 | 0.623 | 0.697 | 0.583 | 0.608 |
| mov4 | 0.759 | 0.783 | 0.969 | 0.786 | 0.743 | 0.770 | 0.730 |
| mov5 | 1.423 | 1.124 | 1.266 | 0.897 | 0.878 | 0.897 | 0.872 |
| mov6 | 1.159 | 1.117 | 1.038 | 0.927 | 0.917 | 0.926 | 0.944 |
| mov7 | 1.180 | 1.169 | 1.009 | 0.910 | 0.917 | 0.909 | 0.870 |
| mov8 | 0.923 | 0.952 | 0.970 | 0.918 | 0.903 | 0.910 | 0.887 |
| mov9 | 0.778 | 0.874 | 0.890 | 0.695 | 0.780 | 0.686 | 0.774 |
| average | 0.972 | 0.974 | 0.988 | 0.820 | 0.807 | 0.805 | 0.792 |
| p-value | **0.038** | **0.000** | 0.571 | 0.059 | **0.015** | **0.014** | **0.004** |
|  |  |  |  |  |  |  |  |
| *wb* |  |  |  |  |  |  |  |
| mov1 | 1.074 | 1.106 | 0.971 | 0.875 | 0.876 | 0.915 | 0.927 |
| mov2 | 0.763 | 0.789 | 0.968 | 0.888 | 0.893 | 0.766 | 0.896 |
| mov3 | 0.996 | 1.059 | 0.941 | 0.878 | 0.942 | 0.859 | 0.944 |
| mov4 | 0.710 | 0.724 | 0.980 | 0.589 | 0.634 | 0.524 | 0.593 |
| mov5 | 1.080 | 1.319 | 0.819 | 0.755 | 0.841 | 0.761 | 0.792 |
| mov6 | 0.721 | 0.801 | 0.900 | 0.740 | 0.782 | 0.716 | 0.707 |
| mov7 | 1.048 | 1.141 | 0.918 | 0.815 | 0.756 | 0.828 | 0.750 |
| average | 0.913 | 0.991 | 0.928 | 0.791 | 0.818 | 0.767 | 0.801 |
| p-value | **0.002** | **0.014** | 0.547 | **0.012** | **0.041** | **0.003** | **0.020** |
|  |  |  |  |  |  |  |  |
| *LanA* |  |  |  |  |  |  |  |
| mov1 | 0.997 | 0.896 | 1.113 | 0.857 | 0.774 | 0.853 | 0.825 |
| mov2 | 1.350 | 1.332 | 1.014 | 0.928 | 0.873 | 0.927 | 0.904 |
| mov3 | 1.278 | 1.172 | 1.090 | 0.856 | 0.832 | 0.869 | 0.859 |
| mov4 | 1.079 | 1.113 | 0.969 | 0.879 | 0.828 | 0.851 | 0.813 |
| mov5 | 1.144 | 1.177 | 0.972 | 0.916 | 0.898 | 0.901 | 0.903 |
| mov6 | 1.007 | 0.984 | 1.023 | 0.840 | 0.782 | 0.786 | 0.771 |
| mov7 | 0.794 | 0.764 | 1.039 | 0.769 | 0.815 | 0.822 | 0.898 |
| mov8 | 0.872 | 0.798 | 1.093 | 0.787 | 0.697 | 0.808 | 0.690 |
| mov9 | 1.108 | 1.098 | 1.009 | 0.889 | 0.882 | 0.900 | 0.864 |
| mov10 | 0.914 | 0.933 | 0.980 | 0.850 | 0.821 | 0.774 | 0.779 |
| average | 1.054 | 1.027 | 1.030 | 0.857 | 0.820 | 0.849 | 0.831 |
| p-value | **0.148** | **0.012** | 0.080 | 0.097 | **0.003** | **0.006** | **0.005** |
